# The Lexicocalorimeter: Gauging public health through caloric input and output on social media

**DOI:** 10.1371/journal.pone.0168893

**Published:** 2017-02-10

**Authors:** Sharon E. Alajajian, Jake Ryland Williams, Andrew J. Reagan, Stephen C. Alajajian, Morgan R. Frank, Lewis Mitchell, Jacob Lahne, Christopher M. Danforth, Peter Sheridan Dodds

**Affiliations:** 1 Department of Mathematics and Statistics, University of Vermont, Burlington, Vermont 05401, United States of America; 2 Vermont Center for Complex Systems, University of Vermont, Burlington, Vermont 05401, United States of America; 3 Computational Story Lab, University of Vermont, Burlington, Vermont 05401, United States of America; 4 Vermont Advanced Computing Core, University of Vermont, Burlington, Vermont 05401, United States of America; 5 School of Information, University of California Berkeley, 102 South Hall #4600, Berkeley, CA 94720, United States of America; 6 Women, Infants and Children, East Boston, Massachusetts 02128, United States of America; 7 Media Lab, Massachusetts Institute of Technology, Cambridge, Massachusetts, 02139, United States of America; 8 School of Mathematical Sciences, North Terrace Campus, The University of Adelaide, Adelaide, South Australia 5005, Australia; 9 Culinary Arts and Food Science, Drexel University, 3141 Chestnut Street, Philadelphia, Pennsylvania 19104, United States of America; Tianjin University of Technology, CHINA

## Abstract

We propose and develop a Lexicocalorimeter: an online, interactive instrument for measuring the “caloric content” of social media and other large-scale texts. We do so by constructing extensive yet improvable tables of food and activity related phrases, and respectively assigning them with sourced estimates of caloric intake and expenditure. We show that for Twitter, our naive measures of “caloric input”, “caloric output”, and the ratio of these measures are all strong correlates with health and well-being measures for the contiguous United States. Our caloric balance measure in many cases outperforms both its constituent quantities; is tunable to specific health and well-being measures such as diabetes rates; has the capability of providing a real-time signal reflecting a population’s health; and has the potential to be used alongside traditional survey data in the development of public policy and collective self-awareness. Because our Lexicocalorimeter is a linear superposition of principled phrase scores, we also show we can move beyond correlations to explore what people talk about in collective detail, and assist in the understanding and explanation of how population-scale conditions vary, a capacity unavailable to black-box type methods.

## Introduction

Online instruments designed to measure social, psychological, and physical well-being at a population level are becoming essential for public policy purposes and public health monitoring [1, 2]. These data-centric gauges both empower the general public with information to allow comparisons of communities at all scales, and naturally complement the broad, established set of more readily measurable socioeconomic indicators such as wage growth, crime rates, and housing prices.

Overall well-being, or quality of life, depends on many factors and is complex to measure [3]. Existing techniques for estimating population well-being range from traditional surveys [1, 4] to estimates of smile-to-frown ratios captured automatically on camera in public spaces [5], and vary widely in the types of data they amass, collection methods, cost, time scales involved, and degree of intrusion. Partly in response to policy makers’ desire for simple “one number” quantification of complex systems—arguably a general human proclivity—many measures are composite in nature. Two examples are (1) the Gallup Well-Being Index, which is based on factors such as life evaluation, emotional health, physical health, healthy behavior, work environment, and basic access to necessary resources [4]; and (2) the Living Conditions measure developed by the United States Census Bureau, which is derived from housing conditions, neighborhood conditions, basic needs met, a “full set” of appliances, and access to help if needed [6].

While such measures will always have their place, we venture that we must resist oversimplification. The dashboard of society should be just that—a rich set of incompatible instruments whose informational content may be observed individually and in total, not unlike the required input needed for flying a plane where knowledge of just a single number representing “things are going well” would be untenable. The construction of data-centric instruments for social systems that deliver more direct, interpretable measures is therefore of great importance as we move forward into the age of ubiquitous (but not complete) measurement.

With the explosive growth of online activity and social media around the world, the massive amount of real-time data created directly by populations of interest has become an increasingly attractive and fruitful source for analysis. Despite the limitation that social media users in the United States are not a random sample of the US population [7], there is a wealth of information in these data sets and uneven sampling can often be accommodated.

Indeed, online activity is now considered by many to be a promising data source for detecting health conditions [8, 9] and gathering public-health information [10, 11], and within the last decade, researchers have constructed a range of online public-health instruments with varying degrees of success. The maturing of these and related instruments along with theoretical models will ultimately fundamentally inform the limits of characterization and predictability of social systems.

In the next two subsections, we cover related research and then describe our approach to measuring the “caloric content” of text.

### Previous work

For a general overview of work relevant to our present effort, we briefly summarize related research concerning public health and well-being in connection with a range of social media and online data sets.

In the difficult realm of predicting pandemics [12], Google Flu Trends [13] enjoyed early success and acclaim. Initially based very simply on search terms, the instrument proved unsurprisingly to be imperfect and in need of a more sophisticated approach [14].

In work by several of the current authors and colleagues, Mitchell *et al.* measured the happiness of tweets across the US and found strong correlations with other indices of well-being at city and state level, such as the Gallup Well-being Index; the Peace Index; the America’s Health Ranking composite index of Behavior, Community and Environment, Policy and Clinical Care metrics; and gun violence (negative correlation) [15]. Using the same instrument in 10 languages, the Hedonometer, we have also shown that the emotional content of tweets tracks major world events [2, 16].

Paul and Dredze found that states with higher obesity rates have more tweets about obesity, and states with higher smoking rates have more tweets about cancer [11]. They also found a negative correlation between exercise and frequency of tweeting about ailments, suggesting “Twitter users are less likely to become sick in states where people exercise.” They further found health care coverage rates to be negatively correlated with likelihood of posting tweets about diseases.

Chunara *et al.* recently found that activity-related interests on Facebook are negatively correlated with being overweight and obese, while interest in television is positively correlated with the same [17].

In an analysis of online recipe queries, West *et al.* found that the number of patients admitted to the emergency room of a major urban hospital in Washington, DC for congestive heart failure (CHF) each month was significantly correlated with average sodium per recipe searched for on the Web in the same month [18].

Eichstaedt and colleagues [19] have demonstrated that psychological language on Twitter outperforms certain composite socioeconomic indices in predicting heart disease at the county level. They were able to show in particular that the expression of negative emotions such as anger on Twitter could be taken as a kind of risk factor at the population scale.

On a US county level, Culotta [20] found that Twitter activity provided a more “fine-grained representation” of community health than demographics alone with the prevalance of particular words that indicate, for example, television habits, or negative engagement.

Finally, in work directly related to our present study, Abbar *et al.* [21] have recently performed a similar analysis of translating food terms used on Twitter into calories. They found a correlation between Twitter calories and obesity and diabetes rates for the US, and explored how food-themed interactions over social networks vary with connectedness, finding suggestions of social contagion. While our approach and results are largely sympathetic, our work incorporates estimates of physical activity which we will show provides essential extra information regarding health; introduces a phrase extraction method we call serial partitioning; and leads to an online implementation, paving the way for a real-time instrument as part of our proposed ‘panometer.’ We also note that we carried out our work concurrently and independently.

### Lexicocalometrics

From the preceding list of studies, it has become clear that we can estimate population-scale levels of health and well-being through social media. Here, we examine the words and phrases people post publicly about food and physical activity on Twitter on a statewide level for the contiguous United States (48 states along with the District of Columbia). As we explain fully below in Estimating Calories from Phrases in the Analysis and Results section, and in Methods and Materials, we group categorically similar words and phrases into lemmas, and we then assign caloric values to these lemmas using the terms and notation “caloric input” for food, *C*_in_, and “caloric output” for activity, *C*_out_. We define the ratio of caloric output to caloric input to be a third quantity, “caloric ratio”:

$$C_{\text{rat}}=\frac{C_{\text{out}}}{C_{\text{in}}}.$$ (1)

While we will focus largely on the three quantities *C*_in_, *C*_out_, and *C*_rat_, we will also explore “caloric difference”, an alternate combination of *C*_in_ and *C*_out_ involving a single parameter:

$$C_{\text{diff}}\left(\alpha \right)=\alpha C_{\text{out}}-\left(1-\alpha \right)C_{\text{in}},$$ (2)

where 0 ≤ *α* ≤ 1. We use “phrase shifts” [2] to show how specific lemmas—e.g., “apples”, “cake with frosting”, “white water rafting”, “knitting”, and “watching tv or movie” contribute to the caloric texture of states across the contiguous US. We then correlate all three values with 37 measures relating to health and well-being, and we find statistically strong correlations with quantities such as high blood pressure, inactivity, diabetes levels, and obesity rates. For ease of language, we will generally speak of phrases rather than lemmas.

We have also generated an accompanying online, interactive instrument for exploring health patterns through the lens of “Twitter calories”: the Lexicocalorimeter. An initial, fixed version of the instrument may be accessed at this paper’s Online Appendices, http://compstorylab.org/share/papers/alajajian2015a/, with a evolvable, production version housed within our larger measurement platform http://panometer.org at http://panometer.org/instruments/lexicocalorimeter (all code for these sites can be found at https://github.com/andyreagan/lexicocalorimeter-appendix). We note that while our online instrument is based on Twitter, it may in principle be used on any sufficiently large text source, social media or otherwise, such as Facebook.

From this point, we structure the core of our paper as follows. In Sec. Analysis and Results, we establish and discuss our findings in depth. Specifically, we: (1) Outline our text analysis of a Twitter corpus from 2011–2012 (see Estimating Calories from Phrases in the Analysis and Results section), reserving full details for Methods and Materials in Sec. Methods and Materials; (2) Present caloric maps of the contiguous US contrasting the 48 states and DC through histograms and phrase shifts (see Caloric Maps of the Contiguous US in Methods and Materials); and (3) Examine how *C*_in_, *C*_in_, *C*_rat_, and *C*_diff_(*α*) correlate with a suite of measures relating to health and well-being. In the Supporting Information, we provide a sample of confirmatory figures as well as all shareable data sets (e.g., IDs for all tweets). We offer concluding thoughts in Concluding Remarks.

## Analysis and results

### Estimating calories from phrases

We used all available geotagged tweets from 2011 and 2012 (around 50 million) from a bounding box of the contiguous US, using Twitter’s garden hose sample (which is a sample of approximately 10% of all tweets, including those that are not geotagged) and the geotag feature to determine from which of the 48 continental states and the District of Columbia each tweet came. From this sample, we counted the total number of times each food and physical activity phrase in our database was tweeted about in each of the 48 continental states and the District of Columbia (see Methods and Materials and the dataset at https://dx.doi.org/10.6084/m9.figshare.4530965.v1 for all tweet IDs). We then used these counts to determine the average caloric input *C*_in_ from food phrase tweets and the average caloric output *C*_out_ from physical activity phrase tweets as follows.

First, we equate each food phrase *s* with the calories per 100 grams of that food, using the notation *C*_in_(*s*). (We also explored serving sizes but the databases available proved far from complete.) We then compute the caloric input for a given text *T* as:

$$C_{\text{in}}\left(T\right)=\frac{\sum_{s\in S_{\text{in}}}C_{\text{in}}\left(s\right)f\left(s|\;T\right)}{\sum_{s}f\left(s|\;T\right)}=\sum_{s\in S_{\text{in}}}C_{\text{in}}\left(s\right)p\left(s|\;T\right),$$ (3)

where *f*(*s*| *T*) is the frequency of phrase *s* in text *T*, *p*(*s*| *T*) is the normalized version, and *S*_in_ is the set of all food phrases in our database.

Second, for each tweeted physical activity phrase, we use an estimate of the Metabolic Equivalent of Tasks, or METs, which we then converted to calories expended per hour, assuming a weight of 80.7 kilograms, the average weight of a North American adult [22]. Analogous to *C*_in_(*T*) above, we then have

$$C_{\text{out}}\left(T\right)=\sum_{s\in S_{\text{out}}}C_{\text{out}}\left(s\right)p\left(s|\;T\right),$$ (4)

where now *S*_out_ is the set of all phrases in our activity database.

We emphasize that both our food and exercise phrase data sets and Twitter databases are necessarily incomplete in nature. The values of *C*_in_ and *C*_out_ are thus not meaningful as absolute numbers but rather have power for comparisons. We also acknowledge that our equivalences are crude—e.g., each mention of a specific food is naively turned into the calories associated with 100 grams of that food—and later on we address our choices in more depth. Nevertheless, our method is pragmatic yet—as we will show—effective, and offers clear directions for future improvement.

For simplicity and ultimately because the results are sufficiently strong, we did not filter tweets beyond their geographic location. Tweets may thus come from individuals, restaurants, sports stores, resorts, news outlets, marketers, fitness apps, tourists, and so on, and further improvements and refinements may be achieved by appropriately constraining the Twitter corpus.

Finally, we take the ratio of *C*_out_(*T*) to *C*_in_(*T*) to obtain the text’s caloric ratio *C*_rat_(*T*). In general, we observe that a higher value of *C*_rat_(*T*) at the population scale would appear to be intuitively better, up to some limit indicating negative energy balance. We note that *C*_rat_ = 1 is not salient and should not be taken to mean a population is ‘balanced calorically’. As we discuss later, using the difference, what we call Caloric Difference, a generalization of *C*_out_ − *C*_in_, generates similar results but, from a framing perspective, we have reservations in creating a scale with a 0 point given the approximate nature of our measures.

### Caloric maps of the contiguous US

We now move to our central analysis and exploration of how our lexicocalorimetric measure varies geographically. We start with visual representations and then continue on to more detailed comparisons.

**Fig 1:**
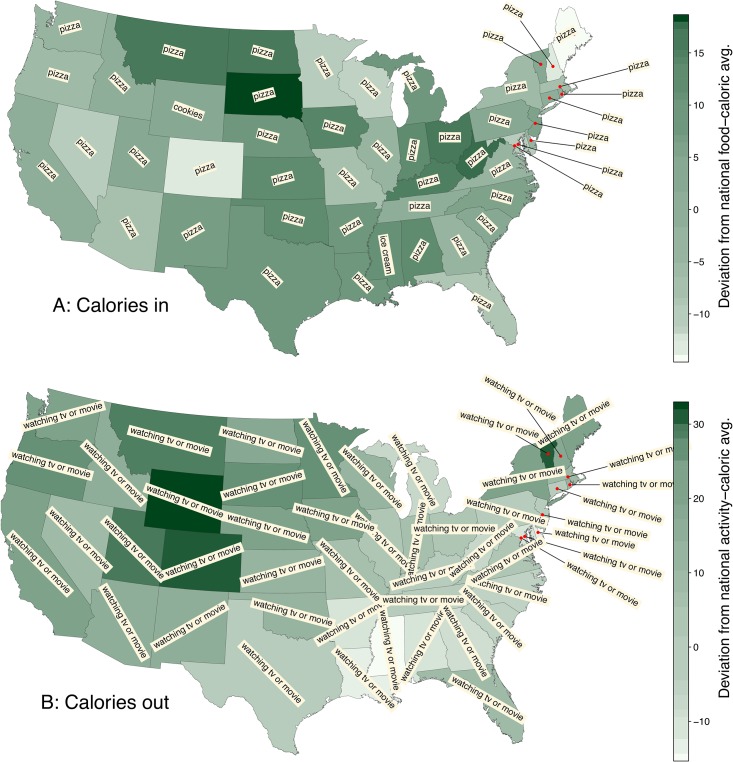
Choropleth maps indicating (A) caloric input *C*_in_ and (B) caloric output *C*_out_ in the contiguous United States (including the District of Columbia) based on 50 million geotagged tweets taken from 2011–2012. For both maps, darker means higher values as per the color bars on the right. The histograms in Fig 5, S2 and S3 Figs show the specific rankings according to these two variables and also *C*_rat_ (see Fig 3). The overlaid phrase lemmas are the most dominant contributors to *C*_in_ and *C*_out_—almost universally “pizza” and “watching tv or movie”.

In Fig 1, we show two choropleth maps of our overall 2011–2012 measures of Twitter’s caloric input *C*_in_ and caloric output *C*_out_. For both maps and those that follow, quantities increase as colors move from light to dark green.

These maps immediately allow for some basic observations which we will delve into and harden up as our analysis proceeds. For the food calories map, we see *C*_in_ is generally largest in the Midwest and the south while Colorado and Maine stand out as states with the lowest calories.

We see a different texture in the activity calories map with the highest caloric output according to our measure appearing in the three-state block of Wyoming, Colorado, and Utah, as well as Vermont. Tweet-based caloric output drops to a low in Mississippi and the surrounding states, while Michigan also appears to have a low value of *C*_out_.

For the food and activities maps in Fig 1, we also show the most dominant phrase for each population’s *C*_in_ and *C*_out_ scores. Almost uniformly, “pizza” (high calorie food) and “watching tv or movie” (low calorie activity) are the lemmas with the largest contributions, a function of both volume and caloric scores. Only Mississippi (“ice cream”) and Wyoming (“cookies”) are exceptions, though “pizza” is still near the top for both.

**Fig 2:**
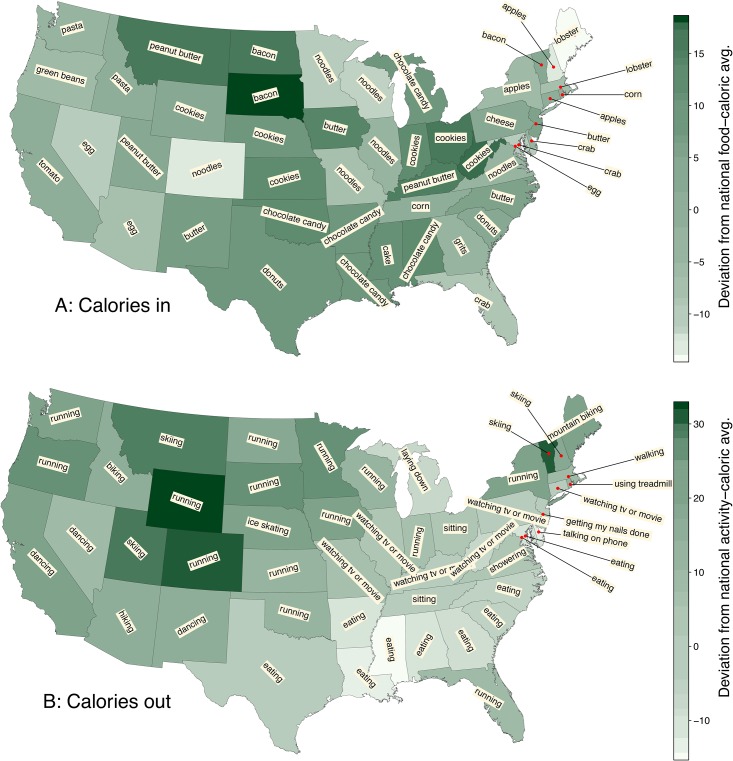
The same choropleth maps for *C*_in_ and *C*_out_ presented Fig 1 but now with phrases whose increased usage contribute the most to a population’s *C*_in_ and *C*_out_ differing from the overall averages of these measures. See the section on Phrase Shifts in Analysis and Results. For example, tweets from Vermont, which was above average for both *C*_in_ and *C*_out_ for 2011–2012, disproportionately contain “bacon” and “skiing”. Michigan was above average for *C*_in_ and below for *C*_out_ in 2011–2012, and the most distinguishing phrases are “chocolate candy” and “laying down”. See Fig 5, S2 and S3 Figs for ordered rankings.

In Fig 2, we present the same choropleth maps from Fig 1, but now with the phrase most distinguishing a population. Specifically, we show phrases whose increased prevalence most contributes to moving a population’s Twitter calorie scores away from the overall average for the contiguous US. For example, if a population’s *C*_in_ is above average, we find the food phrase whose frequency coupled with its caloric content most strongly moves the population’s *C*_in_ up from the average. (We explain in full how we determine these phrases later with phrase shifts in Analysis and Results.) We now see a diverse spread of terms. We find a number of phrases make for reasonable representations:

- “lobster” in Maine and Massachusetts;
- “grits” in Georgia;
- “skiing” in Vermont, New Hampshire, and Utah;
- and “running” in Colorado and a number of other locations.

Prototypical unhealthy foods rise to the top in various states:

- “donuts” in Texas;
- “cake” in Mississippi;
- “chocolate candy” in Louisiana;
- and “cookies” in Indiana.

By contrast, a few “virtuous” foodstuffs appear such as “green beans” in Oregon and “tomato” in California.

Our activity list also includes some rather low intensity ones and we see:

- “eating” rising to the top in Texas, the south, and a number other states;
- “watching tv or movie” in Pennsylvania and elsewhere;
- “sitting” in Tennessee;
- “talking on the phone” in Delaware;
- “getting my nails done” in New Jersey;
- and simply “lying down” in Michigan.

Now, we do not pretend that these phrases all come from individuals diligently recording their present meals or activities. Apart from tweets from individuals, our database contains tweets from companies, advertisers, resorts, and so on. And some phrases are problematic in their generality of meaning, most especially “running” (the word “run” currently has the most meanings in the Oxford English Dictionary). Nevertheless, as we dig deeper into all the phrases found for a particular state, we will continue to find commonsensical lexical patterns.

**Fig 3:**
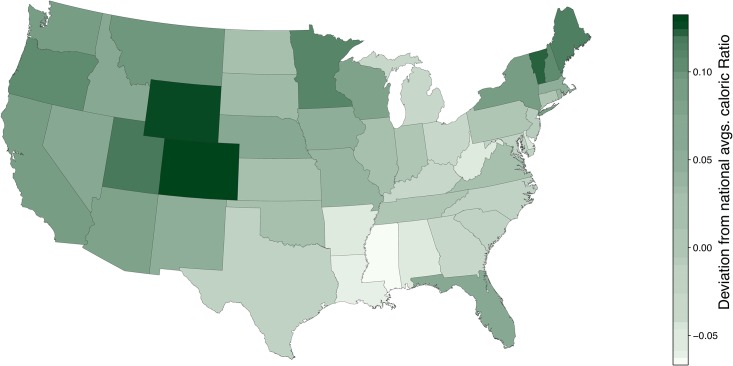
Choropleth for caloric ratio *C*_rat_ = *C*_out_/*C*_in_. See Fig 5, S2 and S3 Figs for ordered rankings.

In Fig 3, we show a choropleth map for caloric ratio, *C*_rat_. We see that the highest values of *C*_rat_ are found in Colorado, Wyoming, and Vermont, and secondarily for Maine, Minnesota, Oregon, and Utah. Low values of *C*_rat_ appear in the region comprising Mississippi, Louisiana, Alabama, and Arkansas, as well as West Virginia.

An initial visual comparison of of Figs 1 and 3, suggest that *C*_out_ is more well aligned with *C*_rat_ than *C*_in_. The reason is that for the present version of the Lexicocalorimeter, *C*_out_ has a larger dynamic range than *C*_in_, roughly 250 to 285 versus 160 to 210 giving ratios of $\frac{210}{160}≃1.31$ and $\frac{285}{250}≃1.14$. We could assert that *C*_in_ is fundamentally less informative but:

1. In Correlations with Other Health and Well-being Measures in our Analysis and Results section, we will find that some measures relating to health and well-being correlate more strongly with *C*_in_ and some with *C*_out_;
2. We may adjust the dynamic range of either measure by rescaling, introducing a kind of tunability [2] to the instrument (a feature we will reserve for future iterations); and
3. Because our food phrase database is a factor of 10 smaller than our activity phrase one, revisions of our instrument may elevate the power of *C*_in_.

**Fig 4:**
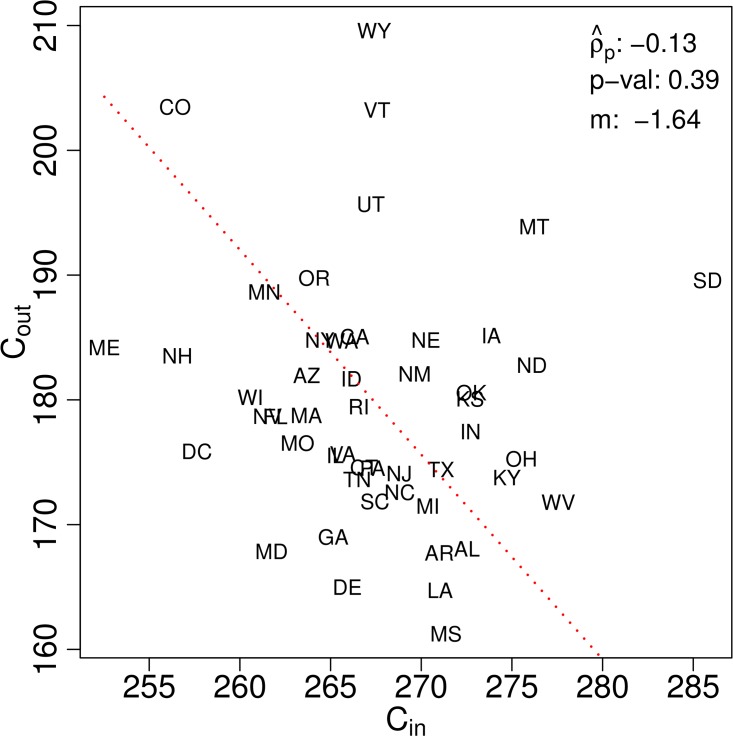
Plots for the contiguous US showing the lack of correlation between caloric input *C*_in_ and caloric output *C*_out_, demonstrating their separate value as they bear different kinds of information. The Pearson correlation coefficient ${\hat{\rho }}_{p}$ is -0.13 and the best line of fit slope is *m* = -1.64. S1 Fig adds plots of *C*_rat_ as a function of *C*_in_ and *C*_out_.

To provide some support for point 1, we compare *C*_out_ and *C*_in_ in Fig 4 (see also S1 Fig). Importantly, we see that the two measures are indeed not well correlated, indicating they contain different kinds of information (Pearson correlation coefficient ${\hat{\rho }}_{p}≃0.13$, *p*-value = 0.39). This demonstrates why we might expect *C*_in_ or *C*_out_ to separately correlate more strongly with other population-level measures, and justifies forming a dashboard using both *C*_in_ and *C*_out_ as well the composite measure of *C*_rat_.

Regarding point 2 above, we have evidently made a number of choices in computing *C*_in_ and *C*_out_ that mean we have already introduced an arbitrary tuning of the ratio *C*_rat_ (e.g., assuming 100 grams of a food and an hour’s worth of activity). Having no principled way of rescaling (i.e., one that is not a function of the data set being studied), we have chosen to leave the measures as computed. As we discuss later, in future iterations we envisage for the Caloric Difference version that introducing tunability of the dynamic ranges of *C*_in_ and *C*_out_—altering the bias of the measure toward food or activity—will allow the Lexicocalorimeter to be refined for a range of purposes such as estimating correlates of diabetes levels versus cancer rates (see Correlations with Other Health and Well-being Measures in Analysis and Results).

### Rankings for the contiguous US

**Fig 5:**
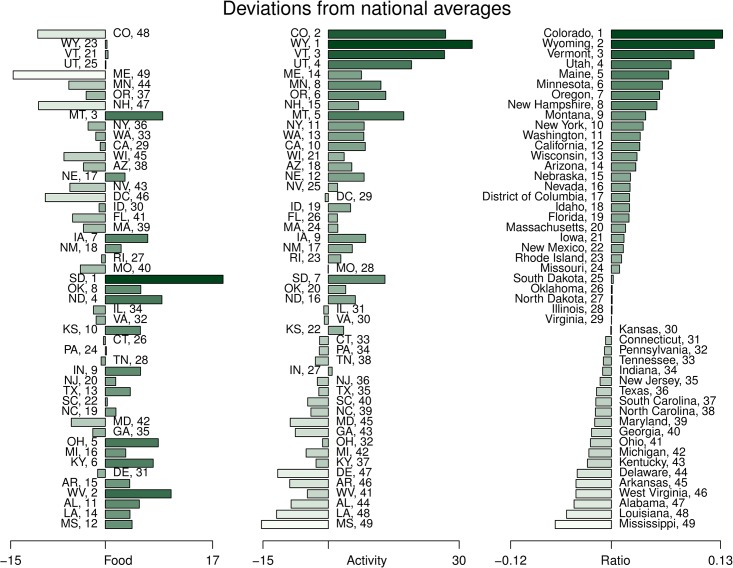
Histograms of caloric intake *C*_in_ (food), caloric output *C*_out_ (activity), and caloric ratio *C*_rat_ for the states of the contiguous US, all ranked by decreasing *C*_rat_. Bars indicate the difference in the three quantities from the overall average with colors corresponding to those used in Figs 1, 2 and 3. We provide the same set of histograms re-sorted by *C*_in_ and *C*_out_ in S2 and S3 Figs.

Having taken in the maps of our three measures *C*_in_, *C*_out_, and *C*_rat_, we now explore the rankings quantitatively, first through the histograms shown in Fig 5. We order the 48 states and DC by *C*_rat_ (rightmost plot) and all bars are relative to the overall average of the specific measure. Numeric rankings for each measure are given next to each bar. In S2 and S3 Figs, we present the same histograms re-sorted respectively by *C*_in_ and *C*_out_.

As was indicated by our inspection the choropleth maps, we do indeed see that *C*_rat_ is more strongly driven by *C*_out_ than *C*_in_ due to the former’s larger dynamic range. The states with the highest values of *C*_rat_ achieve their scores through high levels of *C*_out_ but more variable levels of *C*_in_. Wyoming (23), Vermont (21), and Utah (25) are all middling in *C*_in_ while Colorado (48) and Maine (49) have the lowest ranks for caloric intake. At the trailing end, we see by contrast that low activity ranks are coupled with high ranks for caloric intake.

A few of the more anomalous states are both evident in the *C*_in_ and *C*_out_ histograms and as those appearing farthest away from the best line of fit in the scatter plot of Fig 4. South Dakota has both high values of *C*_in_ and *C*_out_ (ranks of 1 and 7) that arrange to give it a ranking of 25 for *C*_rat_. Maryland ranking 42nd and 45th in *C*_in_ and *C*_out_, is the only state in the ‘bottom’ 10 of both measures.

### Phrase shifts

In our work on measuring happiness, we have developed and extensively used “word shifts” to show which words make a given text appear more positive than another text in aggregate (see [2] and [16]). Such visualizations not only provide our necessary test, but also allow us to draw insight from the lexical tapestry of texts. Here, we will explain and use analogously constructed phrase shifts for both *C*_in_ and *C*_out_ to examine the states at the extremes of our *C*_rat_ rankings, Colorado and Mississippi. Interactive food and activity phrase shifts for the 49 regions of the contiguous US form a central part of our online Lexicocalorimeter: http://panometer.org/instruments/lexicocalorimeter.

We start with two texts: a base “reference text” *T*_ref_, and a “comparison text” *T*_comp_ which we wish to compare to *T*_ref_. In this paper, we will use the Contiguous US as the reference text (weighting the phrase distributions of each state equally), but in principle any text can be used (e.g., in comparing two states, one would be selected as a reference). Our interest is in determining which words or phrases most contribute to or go against the difference in estimated calories. *C*_i/o_(*T*_comp_) − *C*_i/o_(*T*_ref_) where i/o stands for in or out. Following [2] and using Eq (3), we can express the difference as

$$\begin{array}{l} C_{\text{i}/\text{o}}\left(T_{\text{comp}}\right)-C_{\text{i}/\text{o}}\left(T_{\text{ref}}\right)\\ \;=\;\underset{s\in S_{\text{i}/\text{o}}}{\sum }C_{\text{i}/\text{o}}\left(s\right)\left[p(s|T_{\text{comp}})-p(s|T_{\text{ref}})\right]\\ \;=\;\underset{s\in S_{\text{i}/\text{o}}}{\sum }\left[C_{\text{i}/\text{o}}\left(s\right)-C_{\text{i}/\text{o}}^{\left(\text{ref}\right)}\right]\left[p(s|T_{\text{comp}})-p(s|T_{\text{ref}})\right]. \end{array}$$ (5)

We now have a sum contributions due to all phrases. We normalize these contributions as percentages and annotate their structure as follows:

$$\begin{array}{l} \delta C_{i/o}\left(s\right)=\\ \;\frac{100}{\left|C_{i/o}^{\left(\text{comp}\right)}-C_{i/o}^{\left(\text{ref}\right)}\right|}\underset{+/-}{\underset{︸}{\left[C_{i/o}\left(s\right)-C_{i/o}^{\left(\text{ref}\right)}\right]}}\underset{↑/↓}{\underset{︸}{\left[p_{s}^{\left(\text{comp}\right)}-p_{s}^{\left(\text{ref}\right)}\right]}}, \end{array}$$ (6)

where ∑_*s*∈*S*_i/o__ *δC*_i/o_(*s*) = ±100. We use the symbols +/− and ↑/↓ to respectively encode whether the calories of a phrase exceed the average of the reference text, and whether a phrase is being used more or less in the comparison text. We call *δC*_i/o_(*s*) the “per food/activity phrase caloric expenditure shift”. Finally, we sort phrases by the absolute value of *δC*_i/o_(*s*) to create each phrase shift.

**Fig 6:**
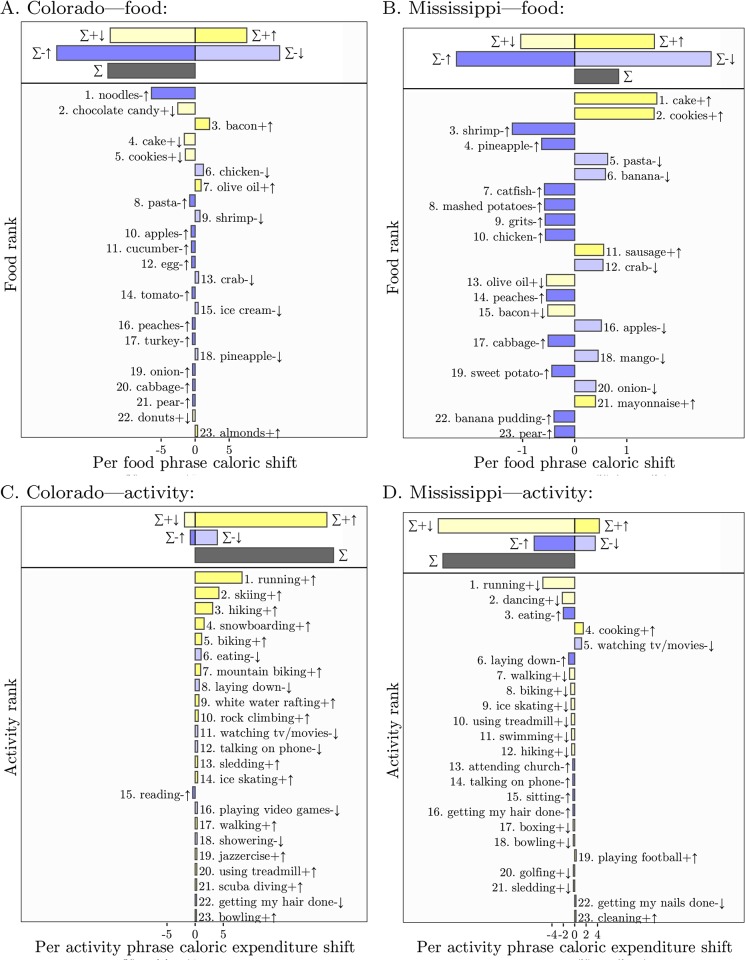
Phrase shifts showing which food phrases and physical activity phrases have the most influence on Colorado and Mississippi’s top and bottom ranking for caloric ratio, when compared with the average for the contiguous United States. Note that phrases are lemmas representing phrase categories. Overall, Colorado scores lower on Twitter food calories (257.4 versus 271.7) and higher on physical activity calories (203.5 versus 161.3) than Mississippi. We provide interactive phrase shifts as part of the paper’s Online Appendices at http://compstorylab.org/share/papers/alajajian2015a/ and at http://panometer.org/instruments/lexicocalorimeter. We explain phrase (word) shifts in the main text (see Eqs 5 and 6), and in full depth in [2] and [16] and online at http://hedonometer.org [23].

In Fig 6, we present food phrase shifts which help to illustrate why:

- Colorado ranks 48/49 for caloric input *C*_in_ (Fig 6A),
- Mississippi ranks 12/49 for caloric input *C*_in_ (Fig 6B),
- Colorado ranks 2/49 for caloric output *C*_out_ (Fig 6C),
- and Mississippi ranks 49/49 for caloric output *C*_out_ (Fig 6D).

These shifts display phrases that fall into four categories:

+↑, yellow: Phrases representing above average quantities (here calories) being used more often. Examples: “cookies” for Mississippi in Fig 6B and “rock climbing” for Colorado in Fig 6C.
-↓, pale blue: Phrases representing below average quantities being used less often. Examples: “watching tv or movie” for Mississippi in Fig 6B and “laying down” for Colorado in Fig 6C.
+↓, pale yellow: Phrases representing above average quantities being used less often. Examples: “chocolate candy” for Colorado in Fig 6A and “running” for Mississippi in Fig 6D.
-↑, blue: Phrases representing below average quantities being used more often. Examples: “reading” for Colorado in Fig 6A and “catfish” for Mississippi in Fig 6B.

Note that depending on the quantity, higher or lower may be “better” and the four categories flip signs in their support. For example, *C*_in_ and *C*_out_ increase with +↑ phrases; after we examine correlations with health and well-being measures in Correlations with Other Health and Well-being measures in Analysis and Results, we will be able to interpret this as “bad” for *C*_in_ and “good” for *C*_out_.

At the top of each phrase shift, the bars indicate the total contribution of each of the four types of phrases, and the black bar the net change. We see that the four net changes arise in different ways.

- Fig 6A: Colorado is lower than average for *C*_in_ largely due to tweeting more about relatively low calorie (per 100 grams) foods: “noodles”, “egg”, “pasta”, and “turkey”. We also find less tweets about high calorie foods such as “candy”, “cake”, and “cookies.” Going against these phrases, we see Colorado does tweet relatively more about “bacon” and “olive oil”, and less about some relatively lower calorie foods “chicken”, “ice cream”, “shrimp”, and “corn”. We note that this does not mean these foods are low calorie in absolute terms (“ice cream” is a good example), just that 100 grams of them are low calorie in comparison to the US baseline.
- Fig 6B: Mississippi almost equally tweets less about a variety of low calorie foods, e.g., “pasta”, “banana”, and “crab” (pale blue bar) while also tweeting more about the complementary range of such foods including “shrimp”, “peaches”, and “pineapple” (dark blue bar). The modest net gain is mostly due to a small increase in tweeting about high calorie foods such as “cake”, “cookies”, and “sausage”.
- Fig 6C: For physical activity, tweets from Colorado show a preponderance of relatively high caloric expenditure phrases (+↑, yellow) including “running”, “skiing”, “hiking”, “snowboarding” and so on. Tweeting less about low effort activities is the only other contribution of any substance—Colorado tweets less about “eating”, “laying down”, and “watching tv or movie”.
- Fig 6D: Mississippi’s low ranking in activity is largely due to tweeting less about high output activities (+↓, pale yellow): less “running”, “dancing”, “walking”, and “biking”. The second most important category is an increase in low output activity phrases such as “eating”, “attending church”, and “talking on the phone.”

In S4, S5, S6 and S7 Figs we complement the four phrase shifts of Fig 6 by showing the top 23 phrases for each of four ways phrases may contribute. Interactive phrase shifts for all of the contiguous US are housed at http://panometer.org/instruments/lexicocalorimeter.

Overall, we find the lexical texture afforded by our phrase shifts is generally convincing, but we expect future improvements in our food and activity data sets will iron out some oddities (we again use the example of ice cream). We also note that phrase shifts are very sensitive and that terms that seem to be being evaluated incorrectly may easily be removed from the phrase set, and that doing so will minimally change the overall score for sufficiently large texts.

### Correlations with other health and well-being measures

We now turn to a suite of statistical comparisons between our three measures—caloric input, caloric output, and caloric ratio—and a collection of demographic, behavioral, health, and psychological quantities.

We use Spearman’s correlation coefficient ${\hat{\rho }}_{s}$ to examine relationships between *C*_in_, *C*_out_, and *C*_rat_ and 37 variables variously relating to food and physical activity, “Big Five” personality traits, and health and well-being rankings (a total of 111 comparisons) [4, 6, 24–33]. To correct for multiple comparisons, we calculate the *q*-value for each correlation coefficient using the Benjamini-Hochberg step-up procedure [34] (the *q*-value is to be interpreted in the same way as a *p*-value). We then consider correlations in reference to the standard significance levels of 0.01 and 0.05.

We must first acknowledge that many of the variables we test against our measures are highly correlated with each other. The food and physical activity-related variables are in the areas of physical activity levels, produce intake and availability rates (including trends in public schools), chronic disease rates, and rates of unhealthy habits. Many of these variables are well known to be influenced by diet and physical activity (e.g., obesity rates [25]), and others may be less directly related (e.g., percent of cropland in each state harvested for fruits and vegetables [28]).

**Fig 7:**
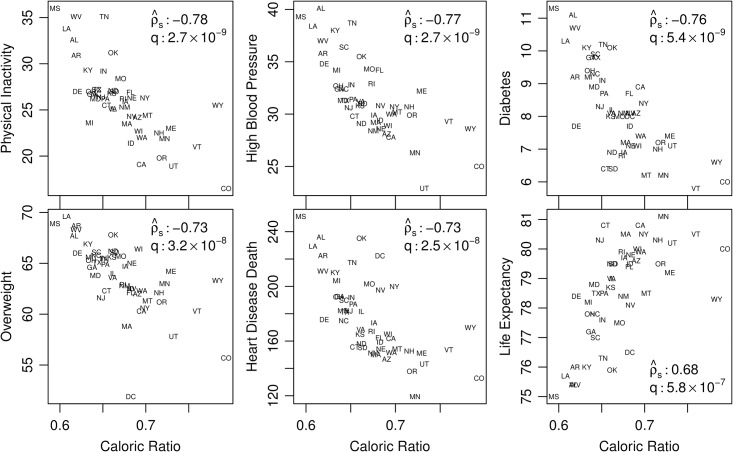
Six demographic quantities compared with caloric ratio *C*_rat_ for the contiguous US. The inset values are the Spearman correlation coefficient ${\hat{\rho }}_{s}$, and the Benjamini-Hochberg *q*-value. See Table 1 for a full summary of the 37 demographic quantities studied here.

To give some grounding for the full set of comparisons, we show in Fig 7 how six demographic quantities vary with caloric ratio *C*_rat_. We see strong correlations with $|{\hat{\rho }}_{s}|\geq 0.68$, and the highest value for Benjamini-Hochberg *q*-value is 5.8×10^ − 7^.

We present a summary of all results in Table 1 where we have ordered and numbered demographic quantities in terms of ascending Benjamini-Hochberg *q*-values for *C*_rat_. For comparison and to further demonstrate the robustness of our approach, in (see S1, S2 and S3 Tables, we reproduce the same analysis with the inclusion of liquids and for a differential measure *C*_diff_(*α*) = *αC*_out_ − (1 − *α*)*C*_in_, both with and without liquids. Here, we choose to set the effective means of *C*_out_ and *C*_in_ equal across the statewide averages (i.e., *α*〈*C*_out_〉 = (1 − *α*)〈*C*_in_〉), resulting in *α* = 0.598. Overall, we find little variation in our results whether we use *C*_rat_ and *C*_diff_(0.598).

**Table 1:** Spearman correlation coefficients, ${\hat{\rho }}_{s}$, and Benjamini-Hochberg *q*-values for caloric input *C*_in_, caloric output *C*_out_, and caloric ratio *C*_rat_ = *C*_out_/*C*_in_ and demographic, data related to food and physical activity, Big Five personality traits [31], health and well-being rankings by state, and socioeconomic status, correlated, ordered from strongest to weakest Spearman correlations with caloric ratio. The two breaks in the table indicate significance levels of 0.01 and 0.05 for the Benjamini-Hochberg *q* of *C*_rat_, corresponding to the first 24 health and/or well-being quantities and then the next four, numbers 25 to 28. The bottom 9 quantities were not significantly correlated with *C*_rat_ according to our tests. S1, S2 and S3 Tables present the same analysis for caloric measures including phrases representing liquids, and for the difference *C*_diff_(*α*) = *αC*_out_ − (1 − *α*)*C*_in_, both without and with liquids included.

Health and/or well-being quantity	${\hat{\rho }}_{s}$ for *C*_rat_	*q*-val	${\hat{\rho }}_{s}$ for *C*_in_	*q*-val	${\hat{\rho }}_{s}$ for *C*_out_	*q*-val
**1.** % no physical activity in past 30 days [24]	-0.78	2.73 × 10^−09^	0.58	5.67 × 10^−05^	-0.66	1.51 × 10^−06^
**2.** % have been physically active in past 30 days [24]	0.78	2.73 × 10^−09^	-0.57	6.53 × 10^−05^	0.67	1.24 × 10^−06^
**3.** % high blood pressure [24]	-0.77	2.73 × 10^−09^	0.32	4.05 × 10^−02^	-0.78	2.73 × 10^−09^
**4.** Adult diabetes rate [25]	-0.76	5.44 × 10^−09^	0.29	6.09 × 10^−02^	-0.77	2.73 × 10^−09^
**5.** CNBC quality of life ranking [26]	-0.76	6.75 × 10^−09^	0.28	7.34 × 10^−02^	-0.77	3.60 × 10^−09^
**6.** % adult overweight/obesity [27]	-0.73	3.16 × 10^−08^	0.55	1.41 × 10^−04^	-0.59	3.07 × 10^−05^
**7.** Heart disease death rate [27]	-0.73	2.50 × 10^−08^	0.34	2.80 × 10^−02^	-0.73	2.30 × 10^−08^
**8.** % adult obesity [25]	-0.72	4.30 × 10^−08^	0.53	2.26 × 10^−04^	-0.59	2.96 × 10^−05^
**9.** Gallup Wellbeing score [4]	0.72	4.69 × 10^−08^	-0.31	4.43 × 10^−02^	0.73	3.99 × 10^−08^
**10.** America’s Health Rankings, overall [24]	-0.72	4.10 × 10^−07^	0.43	4.74 × 10^−03^	-0.67	2.77 × 10^−06^
**11.** Life expectancy at birth [27]	0.68	5.81 × 10^−07^	-0.4	6.91 × 10^−03^	0.65	2.64 × 10^−06^
**12.** % who eat fruit less than once a day [28]	-0.67	1.20 × 10^−06^	0.61	1.39 × 10^−05^	-0.51	5.35 × 10^−04^
**13.** % child overweight/obesity [27]	-0.64	3.53 × 10^−06^	0.27	7.55 × 10^−02^	-0.64	3.20 × 10^−06^
**14.** % who eat vegetables less than once a day [28]	-0.61	1.39 × 10^−05^	0.51	5.33 × 10^−04^	-0.46	1.57 × 10^−03^
**15.** Median daily intake of fruits [28]	0.6	1.98 × 10^−05^	-0.62	8.33 × 10^−06^	0.41	5.37 × 10^−03^
**16.** Smoking rate [27]	-0.59	2.96 × 10^−05^	0.51	5.26 × 10^−04^	-0.48	1.08 × 10^−03^
**17.** Median household income [27]	0.51	5.55 × 10^−04^	-0.53	3.27 × 10^−04^	0.4	8.38 × 10^−03^
**18.** Median daily intake of vegetables [28]	0.5	6.10 × 10^−04^	-0.56	7.44 × 10^−05^	0.31	4.36 × 10^−02^
**19.** % high cholesterol [24]	-0.49	8.11 × 10^−04^	0.23	1.45 × 10^−01^	-0.48	9.05 × 10^−04^
**20.** Brain health ranking [29] (lower is better)	-0.49	8.11 × 10^−04^	0.62	1.39 × 10^−05^	-0.29	5.70 × 10^−02^
**21.** % with bachelor’s degree or higher [6]	0.46	1.57 × 10^−03^	-0.54	1.66 × 10^−04^	0.33	2.82 × 10^−02^
**22.** Colorectal cancer rate [25]	-0.44	4.09 × 10^−03^	0.53	3.59 × 10^−04^	-0.27	8.25 × 10^−02^
**23.** US Census Gini index score [30] (lower is better)	-0.42	5.37 × 10^−03^	-0.03	8.42 × 10^−01^	-0.5	5.55 × 10^−04^
**24.** Avg # poor mental health days, past 30 days [24]	-0.42	5.37 × 10^−03^	0.12	4.80 × 10^−01^	-0.48	1.06 × 10^−03^
**25.** Neuroticism Big Five personality trait [31]	-0.38	1.09 × 10^−02^	0.2	2.03 × 10^−01^	-0.37	1.44 × 10^−02^
**26.** Binge drinking rate [24]	0.37	1.46 × 10^−02^	-0.15	3.56 × 10^−01^	0.41	5.84 × 10^−03^
**27.** Avg # poor physical health days, past 30 days [24]	-0.35	2.34 × 10^−02^	0.19	2.19 × 10^−01^	-0.38	1.13 × 10^−02^
**28.** Farmers markets per 100,000 in pop. [28]	0.34	2.72 × 10^−02^	0.06	7.17 × 10^−01^	0.42	5.14 × 10^−03^
**29.** Strolling of the Heifers locavore score (lower is better) [32]	-0.29	5.86 × 10^−02^	-0.3	5.41 × 10^−02^	-0.45	2.94 × 10^−03^
**30.** Extraversion Big Five personality trait [31]	-0.28	6.94 × 10^−02^	0.03	8.42 × 10^−01^	-0.29	5.63 × 10^−02^
**31.** % schools offering fruit/veg at celebrations [28]	0.24	1.31 × 10^−01^	-0.46	1.96 × 10^−03^	0.05	7.90 × 10^−01^
**32.** Openness Big Five personality trait [31]	0.23	1.31 × 10^−01^	-0.5	6.11 × 10^−04^	0.04	8.10 × 10^−01^
**33.** % cropland harvested for fruits/veg [28]	0.19	2.34 × 10^−01^	-0.62	1.37 × 10^−05^	-0.04	8.10 × 10^−01^
**34.** Conscientiousness Big Five personality trait [31]	-0.12	4.81 × 10^−01^	0.2	2.10 × 10^−01^	-0.05	7.93 × 10^−01^
**35.** % census tracts, healthy food retailer within 1/2 mile [28]	-0.03	8.44 × 10^−01^	-0.52	3.68 × 10^−04^	-0.24	1.31 × 10^−01^
**36.** George Mason overall freedom ranking [33] (lower is freer)	-0.03	8.42 × 10^−01^	-0.11	5.15 × 10^−01^	-0.1	5.64 × 10^−01^
**37.** Agreeableness Big Five personality trait [31]	-0.01	9.61 × 10^−01^	0.22	1.50 × 10^−01^	0.08	6.47 × 10^−01^

Surveying the health-based demographics, we found *C*_rat_ was significantly correlated with all chronic disease-related rates we tested against (high blood pressure (#3), adult diabetes (#4), adult overweight and obesity (#6), heart disease deaths (#7), adult obesity (#8), childhood overweight and obesity (#13), high cholesterol (#19), and colorectal cancer (#22)). All of these but colorectal cancer rate were also significantly correlated with *C*_out_.

Caloric input *C*_in_ results were more mixed. Chronic disease-related rates were also significantly correlated with *C*_in_, with the exception of adult diabetes, childhood overweight and obesity, and high cholesterol, after correcting for multiple comparisons.

The variables relating to unhealthy habits (smoking (#16) and binge drinking rates (#26)) both correlated significantly with all three of our measures with the one exception of binge drinking and caloric input. The direction of correlations for these two habits are opposite each other (e.g., negative for smoking and *C*_rat_, positive for binge drinking and *C*_rat_), consistent with recent work on alcohol consumption [35].

The two variables relating to physical activity rates (percent of population that has had no physical activity in past 30 days (#1), and percent of population that has been physically active in past 30 days (#2)) correlated significantly with all three of our measures. The two measures relating to rates of physical and mental health (average number of poor mental health days in past 30 days (#24), and average number of poor physical health days in past 30 days (#27)) correlated significantly with both *C*_out_ and *C*_rat_, but did not correlate significantly with *C*_in_.

The four variables relating to fruit and vegetable consumption rates all correlated significantly with all three of our measures. The variables relating to presence of produce in the state (percent of cropland in each state harvested for fruits and vegetables (#33), percent of census tracts with a healthy food retailer within one-half mile (#35), and percent of schools offering fruits and vegetables at celebrations (#31)) were significantly correlated with *C*_in_ but were not correlated with *C*_out_ or *C*_rat_. Variables relating to local food (number of farmers markets per 100,000 people (#28) and Strolling of the Heifers locavore score (#29)) were not significantly correlated with *C*_in_, but were significantly correlated with *C*_out_.

Our health and well-being ranking variables included the CNBC quality of life ranking (#5), Gallup Wellbeing ranking (#9), America’s Health Ranking overall state rank (#10), life expectancy ranking (#11), Brain Health ranking (#20), Gini index score (#23), and George Mason’s overall freedom ranking (#36). Caloric ratio correlated with all of these variables except for George Mason’s freedom ranking (which did not correlate with any of our three measures). *C*_out_ correlated significantly with all of these measures except for the Brain Health ranking and the freedom ranking. caloric input *C*_in_ did not correlate significantly with the CNBC quality of life ranking, Gini index score, or freedom ranking.

Regarding correlations with the *Big Five* personality traits, Pesta *et al.* noted that “Neuroticism…emerged as the only consistent Big Five predictor of epidemiologic outcomes (e.g., rates of heart disease or high blood pressure) and health-related behaviors (e.g., rates of smoking or exercise)” [36]. Additionally, “neuroticism correlates with many health-related variables, including depression and anxiety disorders, mortality, coping skill, death from cardiovascular disease, and whether one smokes tobacco” [36]. Here, in keeping with these observations, we found that neuroticism (#25) was indeed the only *Big Five* personality trait that correlated significantly and negatively with caloric ratio.

We also tested our three measures against two measures of socioeconomic status—median income (#17) and percent of state with a bachelor’s degree or higher level of education (#21)—and found these correlations were significant for all three of our measures.

## Concluding remarks

**Fig 8:**
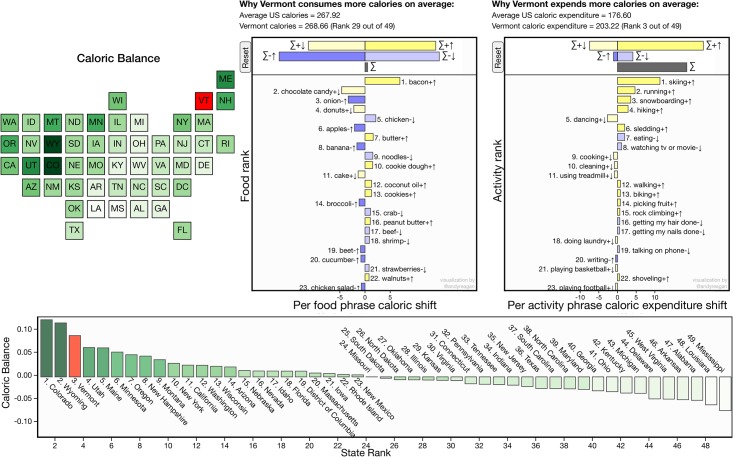
Screenshot of the interactive dashboard for our prototype Lexicocalorimeter site (taken 2015/07/03). An archived development version can be found as part of our paper’s Online Appendices at http://compstorylab.org/share/papers/alajajian2015a/maps.html, and a full dynamic implementation will be part of our Panometer project at http://panometer.org/instruments/lexicocalorimeter. See https://github.com/andyreagan/lexicocalorimeter-appendix for source code.

Our Lexicocalorimeter has thus, when applied to Twitter, proved to find and demonstrate a range of strong, commonsensical patterns and correlations for the contiguous US. We invite the reader to explore our online instrument, a screenshot of which is shown in Fig 8.

Given the complex relationships between health, well-being, happiness, and various measures of socioeconomic status, it is rather difficult to say that we are only measuring health or only measuring well-being. We are also measuring socioeconomic status to some extent. However, the correlations between caloric ratio and measures of socioeconomic status are not as strong as the correlation of caloric ratio with many of the other measures. Given the above, we believe that the caloric content of tweets can be used successfully, along with other well-being and quality of life measures, to help gauge overall well-being in a population.

There are many potential forward directions. A promising avenue is to incorporate tunability to the Lexicocalorimeter by manipulating its dynamic range. While we chose the caloric ratio *C*_rat_ for its generality in the main body of this work, there is more flexibility in the measurement of caloric difference: *C*_diff_(*α*) = *αC*_out_ − (1 − *α*)*C*_in_. Though a universal approach is unclear (*α* should be independent of the particular data set being studied), we may profit from the versatility of *C*_diff_(*α*) when focusing on a single demographic. For example, if we are interested in diabetes rates, we could tune the instrument to obtain the best correlation with known levels, and thereby create a real-time estimator. To do so, we would tune *α* and find the value that gives the highest correlation between *C*_diff_(*α*) and diabetes rates for a given set of populations. Of course, we could use a “black box” method to generate a more optimal fit, but in basing our instrument on food and activity words, we have a far more principled approach that grants us the opportunity not just to mimic but to understand and explain patterns that we find. In particular, our word shifts will be of great use in showing why our hypothetical estimate of diabetes is varying across populations.

We fully recognize that the Twitter population is not the same as the general population; Twitter users differ from the general population in terms of race, age, and urbanity [7]. However, we currently have no reliable way to know, for example, the true age, race, gender, and education level of individual users and as such, are not able to adjust for these factors. While we were able to vet our food and physical activity lists to some extent (as described in Methods and Materials), we could not realistically go through every tweet to be certain that the phrase was being used in the way that we thought. We realize that even if the phrases are being used as we imagine, it does not necessarily mean that the person who tweeted actually performed the physical activity or ate the tweeted-about food (West *et al.* address a similar issue in inferring food consumption from accessing recipes online [18]).

We also currently do not know at what point our metric breaks down at smaller time scales (e.g., months or weeks) or for smaller spatial regions (e.g., city or county) level. Our preliminary research shows that the physical activity metric on its own may be quite effective at the city level, but the food measure may not be accurate on a smaller scale. We have also found the physical activity list to be robust to random partitioning [37], whereas the food list was not. We believe that these preliminary findings may be due to several factors: (a) the size of the food list (just over 1400 phrases) is much smaller than the physical activity phrase list (just over 13,400 phrases); (b) there are generally more tweets about physical activities in our list than the foods in our food list; and (c) the amount of data within a city may not be a large enough sample for any food-based Twitter metric. We note that we have not tried using the metric on counties or Census block or tract groups, and it may be that these are more conducive to the metric.

We propose to use crowdsourcing as a way to build a more comprehensive food phrase list that includes commonly eaten foods with brand names as well as food slang that we did not capture here. Ideally, we would arrive at a food phrase database similar in scale to that of our existing physical activity phrase list. However we move forward, we believe it is clear that the Lexicocalorimeter we have designed and implemented is already of some potency and may be improved substantively in the future.

## Methods and materials

In order to attempt to estimate the “caloric content” of text-extracted phrases [37] relating to food (caloric input) and physical activity (caloric output), we needed comprehensive lists of foods and physical activities and their respective caloric content and expenditure information. Here, we explain in detail how we constructed these phrase lists and assigned calories to each phrase.

In the dataset (https://dx.doi.org/10.6084/m9.figshare.4530965.v1), we provide message IDs for all tweets that are part of our study, and we make both this dataset and other material and visualizations available at the paper’s Online Appendices (http://compstorylab.org/share/papers/alajajian2015a/, and as part of our Panometer project at http://panometer.org/instruments/lexicocalorimeter. We have drawn on Twitter’s Gardenhose API which has been provided to the Computational Story Lab by Twitter.

### Calorie estimates for phrases

We used the USDA National Nutrient Database [38] to approximate the caloric content of foods, and the Compendium of Physical Activities from Arizona State University and the National Cancer Institute [39] to approximate average Metabolic Equivalent of Tasks (METs) for physical activities, which we converted to calories expended per hour of activity [39]. Because the foods listed in the USDA National Nutrient Database are not described in a way that people talk about food, we created a list of food phrases used on Twitter by starting with a kernel of basic food terms from the USDA’s MyPlate website’s food group pages [40]. If the food phrase was not specific, such as “cereal”, we chose the most popular version of that food in the United States via an informal Google search at the time of the study (in this instance, Cheerios). If a brand name food was not in the USDA National Nutrient Database, we chose the closest match we could find. (Please note that this means that data in appendix may be inaccurate when searching brand name items.)

This approach yielded examples of foods in the food groups of fruits, vegetables, grains, proteins, dairy, oils, solid fats, and “empty calories” (e.g., junk food), and built up a list of nearly 1400 food phrases used on Twitter. For the main results we present in this study, we did not include drinks or soups (liquids) in our list. We found there is very little change in our findings when liquids are included, as we discuss below, and we have omitted them at present both for simplicity and because we were not satisfied with a straightforward way of balancing liquid and solid nutrition estimates. Note that we have included ice creams, oils, and some other items that may act as liquids, and these could be separated out for future versions of our instrument.

For physical activity, we used the physical activities listed in the Compendium to build up a list of nearly 14,000 physical activity phrases used on Twitter. The order of magnitude of difference between the length of the two lists exists because of the difference in the number of terms that went into creating each list and the rates at which people tweet about foods vs. physical activities.

### Phrase extraction

A major obstacle to the development of the food and physical activity lists is the determination of those phrases used by individuals that most accurately represent a food or physical activity. Various methods exist which may help one ascertain information about the frequency of usage of higher-order lexical units [37]. However, we require one that not only determines reasonable estimates of frequency of usage, but further, does so with nuance regarding context. For example, one should not count the phrase “apple” as having occurred if it appeared within a larger phrase that was recognized as meaningful, such as “you’re the apple of my eye.” To accomplish these goals, we define a low-assumption text segmentation algorithm, which we refer to as *serial partitioning*.

Serial text partitioning is a greedy algorithm for finding distinct, coherent subsequences (phrases) within a sequence (clause). It relies on the directionality of a sequence, and so is particularly adept for processing text into multi-word expressions for many modern languages. The algorithm relies on an objective function, which we will generally refer to as L. At a high level, the algorithm seeks to find find the largest subsequences possible, following a chain of optimizing, growing subsequences.

In the context of this article, we define L relative to a text *T* as follows, providing pseudocode below. First, let $f:S\to R^{\geq 0}$ be the random partition frequency function [37] under the pure random partition probability ($q=\frac{1}{2}$) for the text *T*. We then apply the model of context developed in [41] under the parameterization *q* = 1, so that a given phrase *s* is a member of *ℓ*(*s*) contexts $C_{s}$ (e.g., the phrase *s* = (*New*, *York*, *City*) is a member of three contexts, labeled $C_{s}=\{\left(*,York,City),(New,*,City),\text{ and }(New,York,*)\}\right)$. Then for $C\in C_{s}$, we consider the context-local likelihood probabilities:

$$P\left(s|C\right)=\frac{f(s)}{\sum_{t\in C}f\left(t\right)},$$ (7)

and prescribe to *s* the likelihood-minimizing context

$$C_{s}=\underset{C\in C_{s}}{\text{argmin}}\left(P\left(s|C\right)\right),$$ (8)

which chooses the context-pattern that is most prevalent in *T*. The objective function for this instantiation of serial partitioning is then defined as

$$L\left(s\right)=P\left(s|\;C_{s}\right),$$ (9)

and referred to as the *local likelihood* of a phrase *s*.

An outline of serial text partitioning of a (left-to-right) directional clause, given an objective function $L:S\to R^{\geq 0}$ (whose maximization is desired, in this case) that is zero on the empty phrase (⋅), and a clause *t* = (*t*_1_, ⋯, *t*_*ℓ*(*t*)_), consisting of *ℓ*(*t*) words is as follows:

1: **procedure** SerialTextPartitioning(t)

2:  P←(·)                     ⊳ init. the partition.

3:  *s* ← (⋅)                       ⊳ init. the phrase.

4:  **for** *i* ∈ (1, ⋯, *ℓ*(*t*)) **do**

5:   **if** $L\left(s^{⌢}t_{i}\right)>L\left(s\right)$ **then**

6:    *s* ← *s*^⌢^ *t*_*i*_

7:   **else**

8:    $P\leftarrow P^{⌢}s$

9:    *s* ← *t*_*i*_

10:  **return** P

Note that for any *a*, *b* ∈ *S*, *a* ⌢ *b* = (*a*_1_, ⋯, *a*_*ℓ*(*a*)_, *b*_1_, ⋯*b*_*ℓ*(*b*)_) denotes the concatenation of phrases, and that for convenience, a single sequence element, *a*_*i*_, may be treated as sequence of one term, (*a*_*i*_).

We manually applied the following criteria for constructing both food and exercise phrase lists. For a phrase to be included, it had to be a phrase that used the food or physical activity word(s) in a way that pertained to eating or physical activity; we excluded phrases that were part of hashtags, Twitter user names, song lyrics, or names of organizations or businesses, and phrases that appeared four or fewer times were not included. Misspellings and alternate spellings were included if we happened upon them (for example, “mash potatoes” instead of “mashed potatoes”), but we did not go out of our way to search for them. We queried questionable phrases to be sure that the majority of their uses were referring to the item of interest. Because we were building up from a small list, some specific versions of foods were included while more general forms were not. For example, because we built phrases up from “strawberry,” “strawberry jam” was included while we did not conduct a larger search for “jam”. In another example, in building phrases up from “bacon,” “bacon wrapped dates” turned up so we included those dates but did not conduct a larger search for all possible “dates”. (Note: We removed the physical activities category ‘sexual activity’ from the study because the task of determining meaning and context was too difficult.)

We searched for phrases containing the physical activities in multiple tenses in order to capture as much information as possible. For example, for the activity type *shoveling snow*, we searched for the forms of shovel, shovel*ing*, and shovel*ed*. Tweets were initially converted to all lowercase text, so we were assured that we were not missing data due to capitalization. To match each food phrase with its closest caloric data, we found the most closely corresponding food from the USDA National Nutrient Database, counting all vegetables and fruits in their raw form unless the phrase indicated otherwise. Similarly, we entered meats as roasted or cooked with dry heat, not fried, unless the phrase indicated otherwise or there was no homemade option. We used the nutrition content of homemade versions of foods (for example, baked goods) rather than store-bought foods unless the phrase indicated otherwise. Our approach, while systematic, was not exhaustive, nor is it the only way of taking on this challenge; there are certainly other methods that we expect to yield similar results.

Finally, we lemmatized the food phrases by their code in the USDA National Nutrient Database. If there were food phrases that were more general in each set of phrases that held the same code, we used the more general phrase as the lemma.

We lemmatized the activity phrases by their METs and activity category. Activity categories were largely the same as listed in the Compendium with slight changes due to items in Compendium being listed in a Miscellaneous category, etc. This yielded instances of physical activity phrases that were in the same activity category but were very different with the same METs being included in the same lemma. From this level of lemmatization, we then used our best judgement to break these lemmas down further until proper phrases were included in each lemma.

## Supporting information

S1 Fig Plots for the contiguous US showing the relationships *C*_rat_ versus *C*_in_ (left), and *C*_rat_ versus *C*_out_ (right). With its larger range, caloric output *C*_out_ is more tightly coupled with the ratio *C*_rat_.
(TIFF)

S2 Fig Histograms as per Fig 5 with states sorted by food rank. The bar colors correspond those used in for the choropleth maps in Figs 1, 2 and 3.
(TIFF)

S3 Fig Histograms as per Fig 5 with states sorted by activity rank. The bar colors correspond those used in for the choropleth maps in Figs 1, 2 and 3.
(TIFF)

S4 Fig Food phrase shifts for Colorado, broken down into the four ways phrases may contribute to a shift. See Fig 6A for the combined shift. See Phrase Shifts in the Analysis and Results section for an explanation of phrase shifts.
(TIFF)

S5 Fig Food phrase shifts for Mississippi, broken down into the four ways phrases may contribute to a shift. See Fig 6B for the combined shift. See Phrase Shifts in the Analysis and Results section for an explanation of phrase shifts.
(TIFF)

S6 Fig Activity phrase shifts for Colorado, broken down into the four ways phrases may contribute to a shift. See Fig 6C for the combined shift. See Phrase Shifts in the Analysis and Results section for an explanation of phrase shifts.
(TIFF)

S7 Fig Activity phrase shifts for Mississippi, broken down into the four ways phrases may contribute to a shift. See Fig 6D for the combined shift. See Phrase Shifts in the Analysis and Results section for an explanation of phrase shifts.
(TIFF)

S1 TableIdentical to Table 1 but with liquids included.Spearman correlation coefficients, ${\hat{\rho }}_{s}$, and Benjamini-Hochberg *q*-values for caloric input *C*_in_, caloric output *C*_out_, and caloric ratio *C*_rat_ = *C*_out_/*C*_in_ and demographic data related to food and physical activity, Big Five personality traits [31], health and well-being rankings by state, and socioeconomic status, correlated, ordered from strongest to weakest Spearman correlations with caloric ratio.(PDF)

S2 TableIdentical to Table 1 but using a caloric difference rather than caloric ratio.Spearman correlation coefficients, ${\hat{\rho }}_{s}$, and Benjamini-Hochberg *q*-values for caloric input *C*_in_, caloric output *C*_out_, and caloric difference *C*_diff_(*α*) = *αC*_out_ + (1 − *α*)*C*_in_ and demographic data related to food and physical activity, Big Five personality traits [31], health and well-being rankings by state, and socioeconomic status, correlated, ordered from strongest to weakest Spearman correlations with caloric ratio. We chose *α* so that the average of *C*_out_ matched the average of *αC*_in_.(PDF)

S3 TableIdentical to Table 1 but including liquids and using a caloric difference rather than caloric ratio.Spearman correlation coefficients, ${\hat{\rho }}_{s}$, and Benjamini-Hochberg *q*-values for caloric input *C*_in_, caloric output *C*_out_, and caloric difference *C*_diff_(*α*) = *αC*_out_ + (1 − *α*)*C*_in_ and demographic data related to food and physical activity, Big Five personality traits [31], health and well-being rankings by state, and socioeconomic status, correlated, ordered from strongest to weakest Spearman correlations with caloric ratio. We chose *α* so that the average of *C*_out_ matched the average of *αC*_in_.(PDF)

## References

[1] Health-related quality of life: Well-being concepts; 2013. Health-related quality of life: Well-being concepts. http://www.cdc.gov/hrqol/wellbeing.htm; Accessed March 29, 2014.

[2] Dodds PS, Harris KD, Kloumann IM, Bliss CA, Danforth CM. Temporal patterns of happiness and information in a global social network: Hedonometrics and Twitter. PLoS ONE. 2011;6:e26752 Draft version available at http://arxiv.org/abs/1101.5120v4. Accessed November 15, 2014. 10.1371/journal.pone.0026752 22163266 PMC3233600

[3] Diener E, Diener M, Diener C. Factors predicting the subjective well-being of nations. Journal of Personality and Social Psychology. 1995;69:851–864. 7473035 10.1037//0022-3514.69.5.851

[4] State of the States. http://www.gallup.com/poll/125066/State-States.aspx; Accessed March 29, 2014.

[5] Stimmungsgasometer. http://xn--fhlometer-q9a.de/; Accessed March 29, 2014.

[6] Siebens J. Extended measures of well-being: Living conditions in the United States: 2011; 2013. Accessed on March 15, 2014. Available from: http://www.census.gov/prod/2013pubs/p70-136.pdf.

[7] Duggan M, Brenner J. The demographics of social media users—2012; 2013. Accessed on March 15, 2014. Available from: http://www.pewinternet.org/files/old-media//Files/Reports/2013/PIP_SocialMediaUsers.pdf.

[8] Signorini A, Segre AM, Polgreen PM. The use of Twitter to track levels of disease activity and public concern in the US during the influenza A H1N1 pandemic. PLoS ONE. 2011;6:e19467 10.1371/journal.pone.0019467 21573238 PMC3087759

[9] Prieto VM, Matos S, Alvarez M, Cacheda F, Oliveira JL. Twitter: A good place to detect health conditions. PLoS ONE. 2014;9:e86191 10.1371/journal.pone.0086191 24489699 PMC3906034

[10] Chew C, Eysenbach G. Pandemics in the age of Twitter: Content analysis of tweets during the 2009 H1N1 outbreak. PLoS ONE. 2010;5:e14118 10.1371/journal.pone.0014118 21124761 PMC2993925

[11] Paul MJ, Dredze M. You are what you tweet: Analyzing Twitter for public health. ICWSM. 2011;20:265–272.

[12] Watts DJ, Muhamad R, Medina D, Dodds PS. Multiscale, resurgent epidemics in a hierarchcial metapopulation model. Proc Natl Acad Sci. 2005;102(32):11157–11162. 10.1073/pnas.0501226102 16055564 PMC1183543

[13] Google Flu Trends, https://www.google.org/flutrends/; accessed March 1, 2015.

[14] Lazer D, Kennedy R, King G, Vespignani A. The parable of Google Flu: Traps in Big Data analysis. Science Magazine. 2014;343:1203–1205. 10.1126/science.1248506 24626916

[15] Mitchell L, Frank MR, Dodds PS, Danforth CM. The Geography of Happiness: Connecting Twitter sentiment and expression, demographics, and objective characteristics of place. PLoS ONE. 2013;8:e64417 10.1371/journal.pone.0064417 23734200 PMC3667195

[16] Dodds PS, Clark EM, Desu S, Frank MR, Reagan AJ, Williams JR, et al Human language reveals a universal positivity bias. Proc Natl Acad Sci. 2015;112(8):2389–2394. 10.1073/pnas.1411678112 25675475 PMC4345622

[17] Chunara R, Bouton L, Ayers JW, Brownstein JS. Assessing the online social environment for surveillance of obesity prevalence. PLoS ONE. 1995;8:e61373 10.1371/journal.pone.0061373 PMC3634787 23637820

[18] West R, White RW, Horvitz E. From cookies to cooks: Insights on dietary patterns via analysis of web usage logs. In: Proceedings of the 22nd international conference on World Wide Web. ACM; 2013. p. 1399–1410.

[19] Eichstaedt JC, Schwartz HA, Kern ML, Park G, Labarthe DR, Merchant RM, et al. Psychological language on Twitter predicts county-level heart disease mortality. Psychological Science. 2015;. 10.1177/0956797614557867 PMC4433545 25605707

[20] Culotta A. Estimating County Health Statistics with Twitter. In: Proceedings of the 32Nd Annual ACM Conference on Human Factors in Computing Systemes. CHI’14. New York, NY, USA: ACM; 2014. p. 1335–1344. Available from: http://doi.acm.org/10.1145/2556288.2557139.

[21] Abbar S, Mejova Y, Weber I. You tweet what you eat: Studying food consumption through Twitter. New York, NY, USA: ACM; 2015 Available from: http://doi.acm.org/10.1145/2702123.2702153.

[22] Walpole SC, Prieto-Merino D, Edwards P, Cleland J, Stevens G, Roberts I. The weight of nations: an estimation of adult human biomass. BMC Public Health. 2012;12:439 10.1186/1471-2458-12-439 22709383 PMC3408371

[23] Hedonometer 2.0: Measuring happiness and using word shifts; Computational Story Lab blog; October 6, 2014; http://compstorylab.org/2014/10/06/hedonometer-2-0-measuring-happiness-and-using-word-shifts/; Accessed on March 1, 2015.

[24] Americas Health Rankings report—State Health Statistics; http://AmericasHealthRankings.org, Accessed March 15, 2014.

[25] Centers for Disease Control and Prevention; http://www.cdc.gov, Accessed March 15, 2014.

[26] CNBC overall rankings 2012; http://www.cnbc.com/id/100016697, Accessed March 15, 2014.

[27] State Health Facts—The Henry J. Kaiser Family Foundation; http://kff.org/statedata, Accessed March 15, 2014.

[28] State indicator report on fruits and vegetables. National Center for Chronic Disease Prevention and Health Promotion, Division of Nutrition, Physical Activity, and Obesity. Centers for Disease Control and Prevention, US Department of Health and Human Services, 2013; http://www.cdc.gov/nutrition/downloads/State-Indicator-Report-Fruits-Vegetables-2013.pdf, Accessed March 15, 2014.

[29] America’s Brain Health Index; http://www.beautiful-minds.com/AmericasBrainHealthIndex, Accessed March 15, 2014.

[30] US Census American FactFinder; http://factfinder2.census.gov/faces/nav/jsf/pages/index.xhtml, Accessed March 15, 2014.

[31] Rentfrow PJ, Gosling SD, Jokela M, Stillwell DJ, Kosinski M, Potter J. Divided we stand: Three psychological regions of the United States and their political, economic, social, and health correlates. Journal of Personality and Social Psychology. 2013;105(6):996–1012. 10.1037/a0034434 24128185

[32] Strolling of the Heiders Locavore Index; http://www.strollingoftheheifers.com/locavoreindex/, Accessed March 15, 2014.

[33] Freedom in the 50 states, Mercatus Center, George Mason University; http://freedominthe50states.org/, Accessed March 15, 2014.

[34] Benjamini Y, Hochberg Y. Controlling the false discovery rate: A practical and powerful approach to multiple testing. Journal of the Royal Statistical Society Series B (Methodological). 1995;57:289–300.

[35] French MT, Popovici I, Maclean JC. Do alcohol consumers exercise more? Findings from a national survey. Am J Health Promot. 2009;24:2–10. 10.4278/ajhp.0801104 19750956 PMC2747097

[36] Pesta BJ, Bertsch S, McDaniel MA, Mahoney CB, Poznanski PJ. Differential epidemiology: IQ, neuroticism, and chronic disease by the 50 U.S. states. Intelligence. 2012;40:107–114. 10.1016/j.intell.2012.01.011

[37] Williams JR, Lessard PR, Desu S, Clark EM, Bagrow JP, Danforth CM, et al Zipf’s law holds for phrases, not words. Nature Scientific Reports. 2015;5:12209 Available online at http://arxiv.org/abs/1406.5181. 10.1038/srep12209 PMC4531284 26259699

[38] U.S. Department of Agriculture, Agricultural Research Service, USDA National Nutrient Database for Standard Reference, release 25; 2013; http://www.ars.usda.gov/ba/bhnrc/ndl; Accessed March 15, 2014.

[39] Ainsworth BE, Haskell WL, Herrmann SD, Meckes N, Bassett DR Jr, Tudor-Locke C, et al The Compendium of Physical Activities Tracking Guide. Healthy Lifestyles Research Center, College of Nursing & Health Innovation, Arizona State University; 2013.

[40] USDA MyPlate food groups; http://www.choosemyplate.gov/food-groups/; Accessed May 15, 2015.

[41] Williams JR, Clark EM, Bagrow JP, Danforth CM, Dodds PS. Identifying missing dictionary entries with frequency-conserving context models. Physical Review E. 2015;92:042808 10.1103/PhysRevE.92.042808 26565290

