# Supplementary figures and images for: The Lexicocalorimeter: Gauging public health through caloric input and output on social media
Source: PLoS One. 2017 Feb 10;12(2):e0168893. doi: 10.1371/journal.pone.0168893 (PMC5302853; doi:10.1371/journal.pone.0168893)

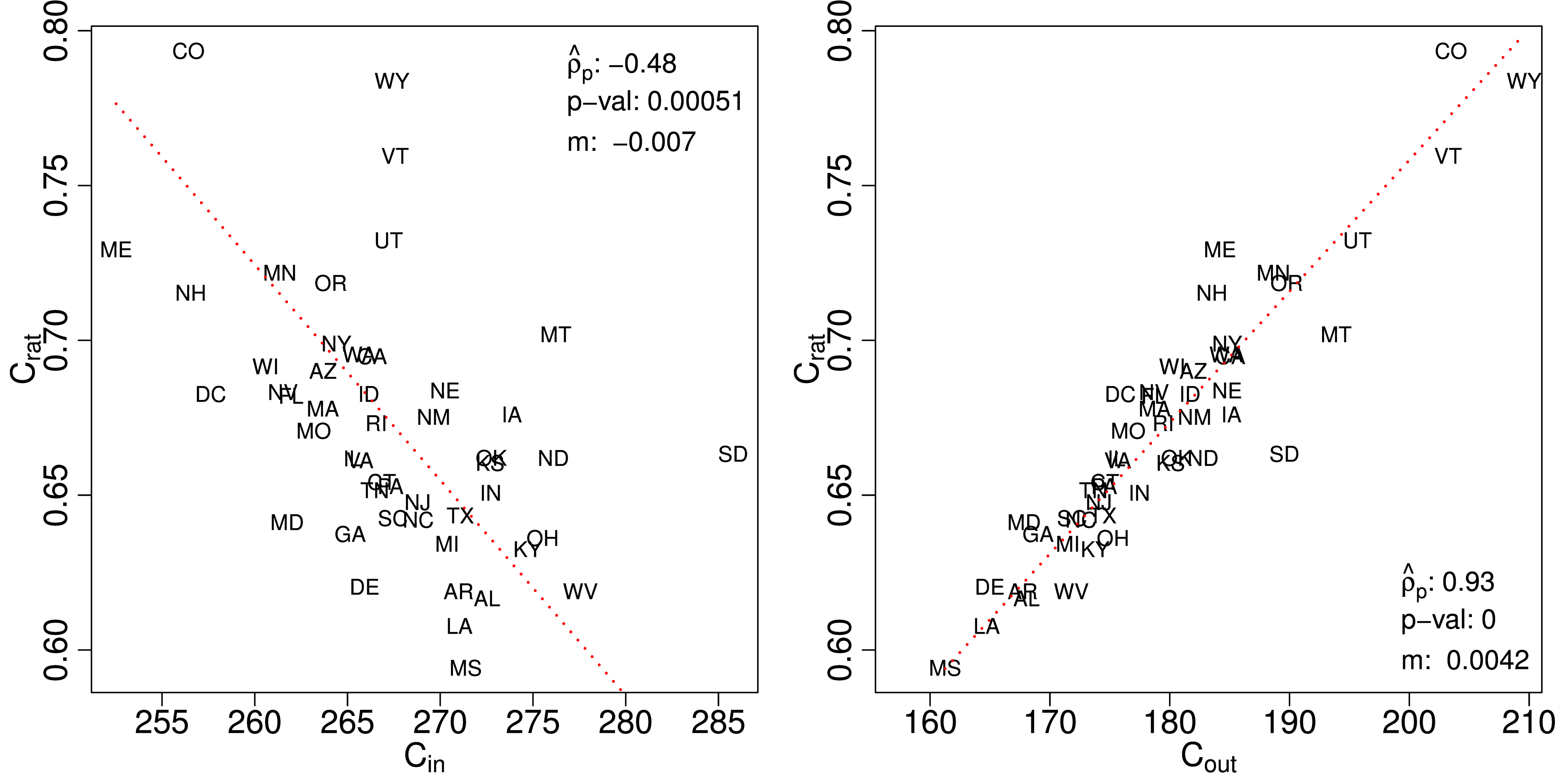

Supplement: S1 Fig — With its larger range, caloric output Cout is more tightly coupled with the ratio Crat. (TIFF) [file pone.0168893.s001.tiff]

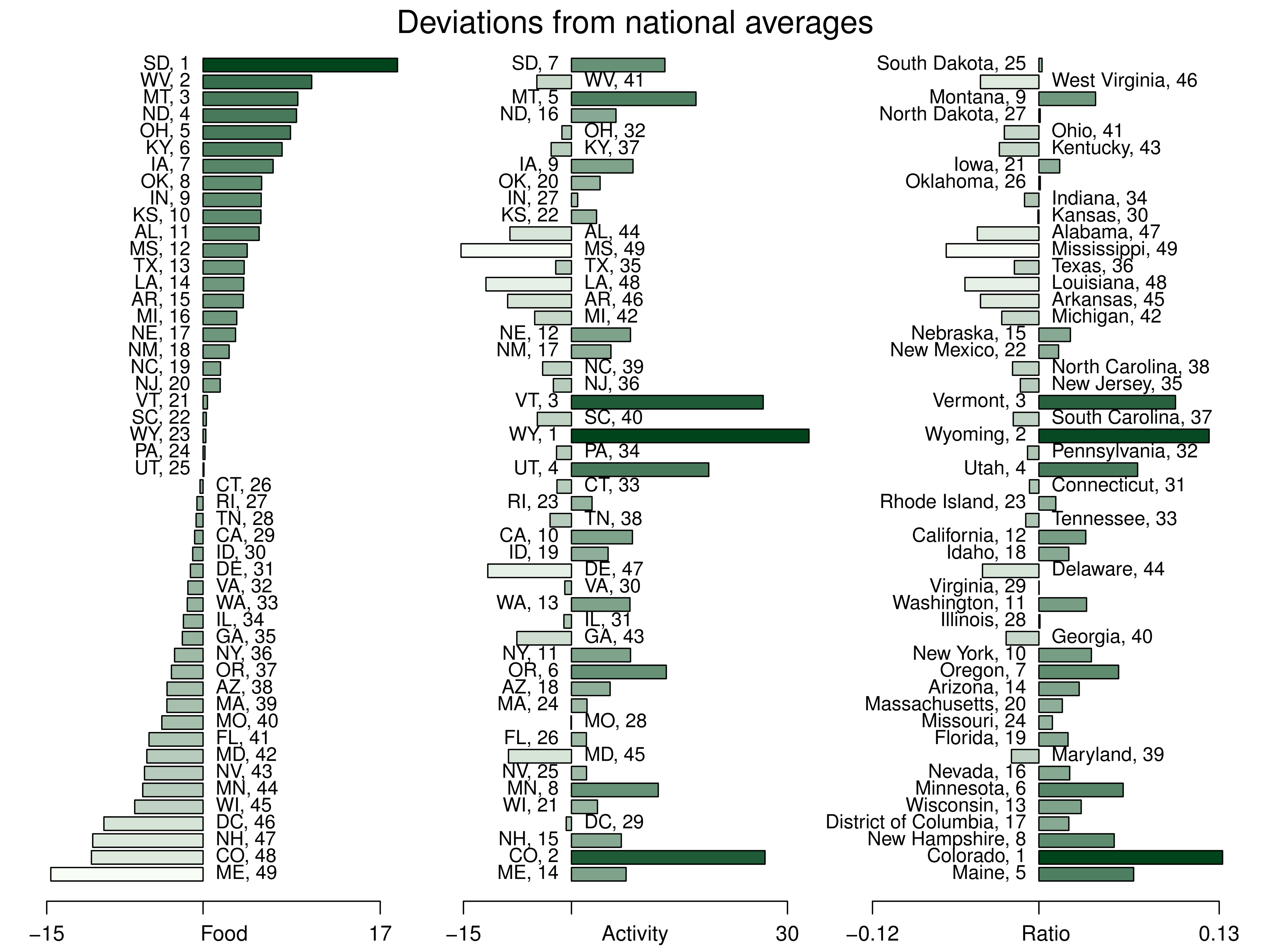

Supplement: S2 Fig — The bar colors correspond those used in for the choropleth maps in Figs 1, 2 and 3. (TIFF) [file pone.0168893.s002.tiff]

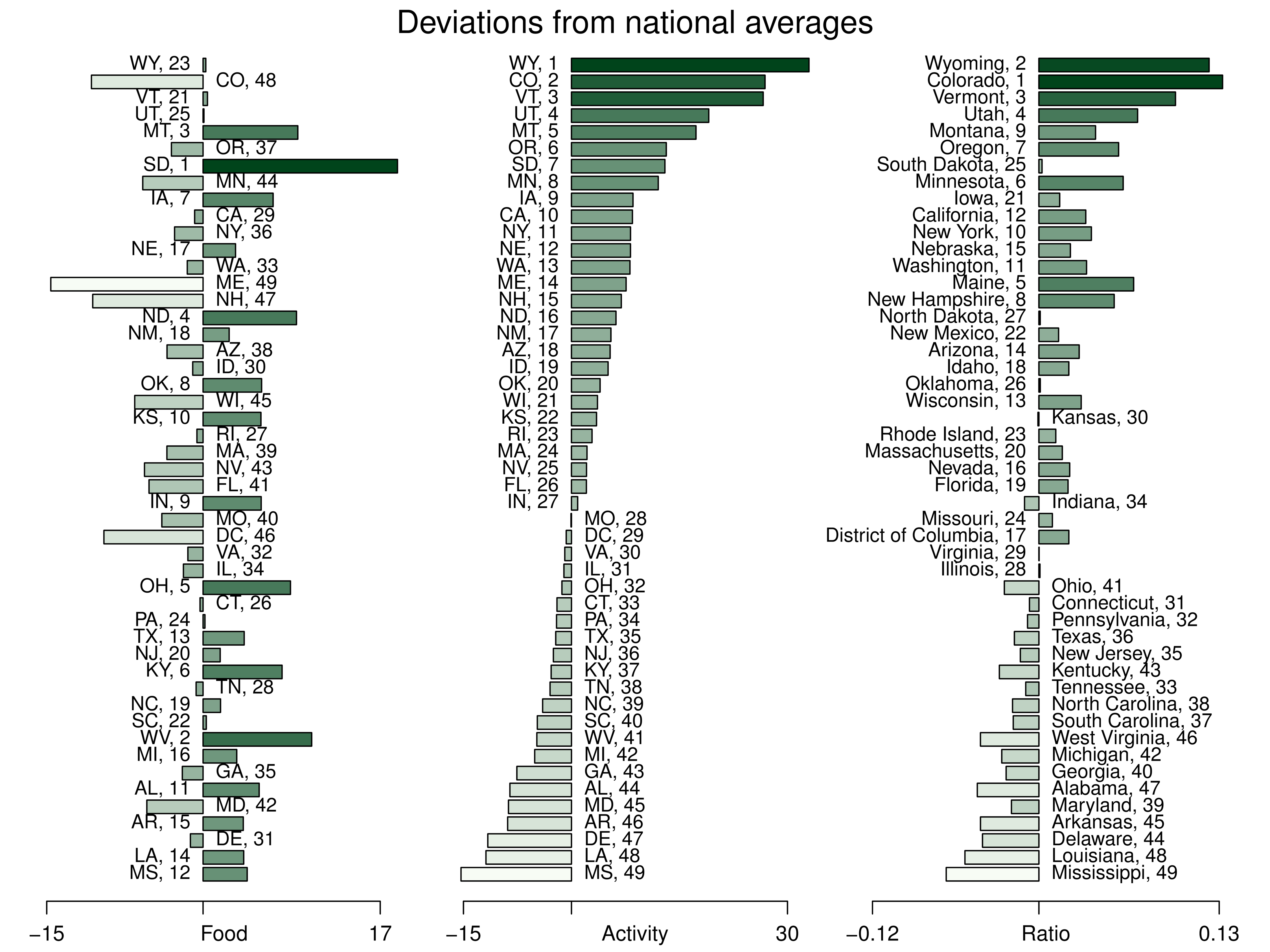

Supplement: S3 Fig — The bar colors correspond those used in for the choropleth maps in Figs 1, 2 and 3. (TIFF) [file pone.0168893.s003.tiff]

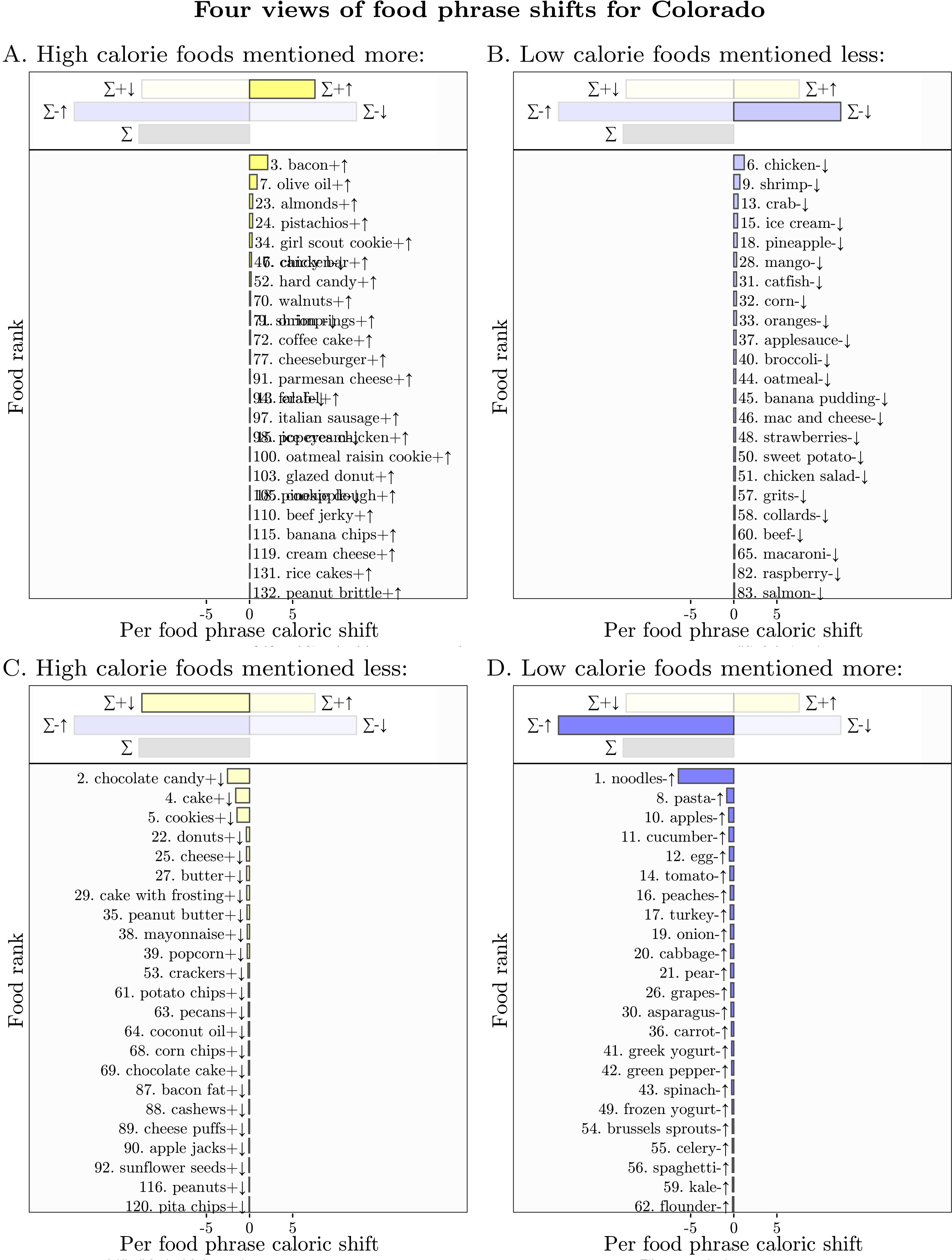

Supplement: S4 Fig — See Fig 6A for the combined shift. See Phrase Shifts in the Analysis and Results section for an explanation of phrase shifts. (TIFF) [file pone.0168893.s004.tiff]

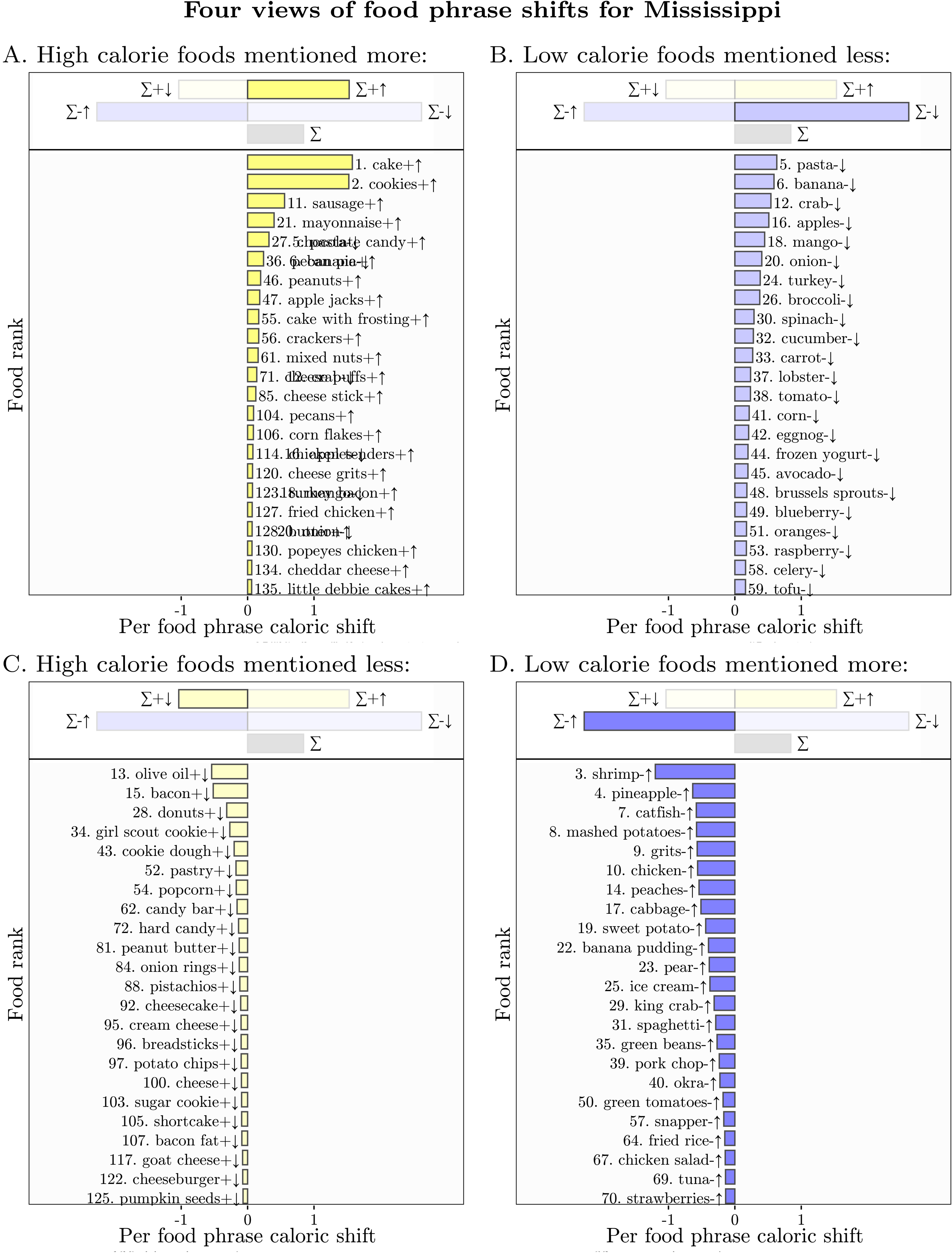

Supplement: S5 Fig — See Fig 6B for the combined shift. See Phrase Shifts in the Analysis and Results section for an explanation of phrase shifts. (TIFF) [file pone.0168893.s005.tiff]

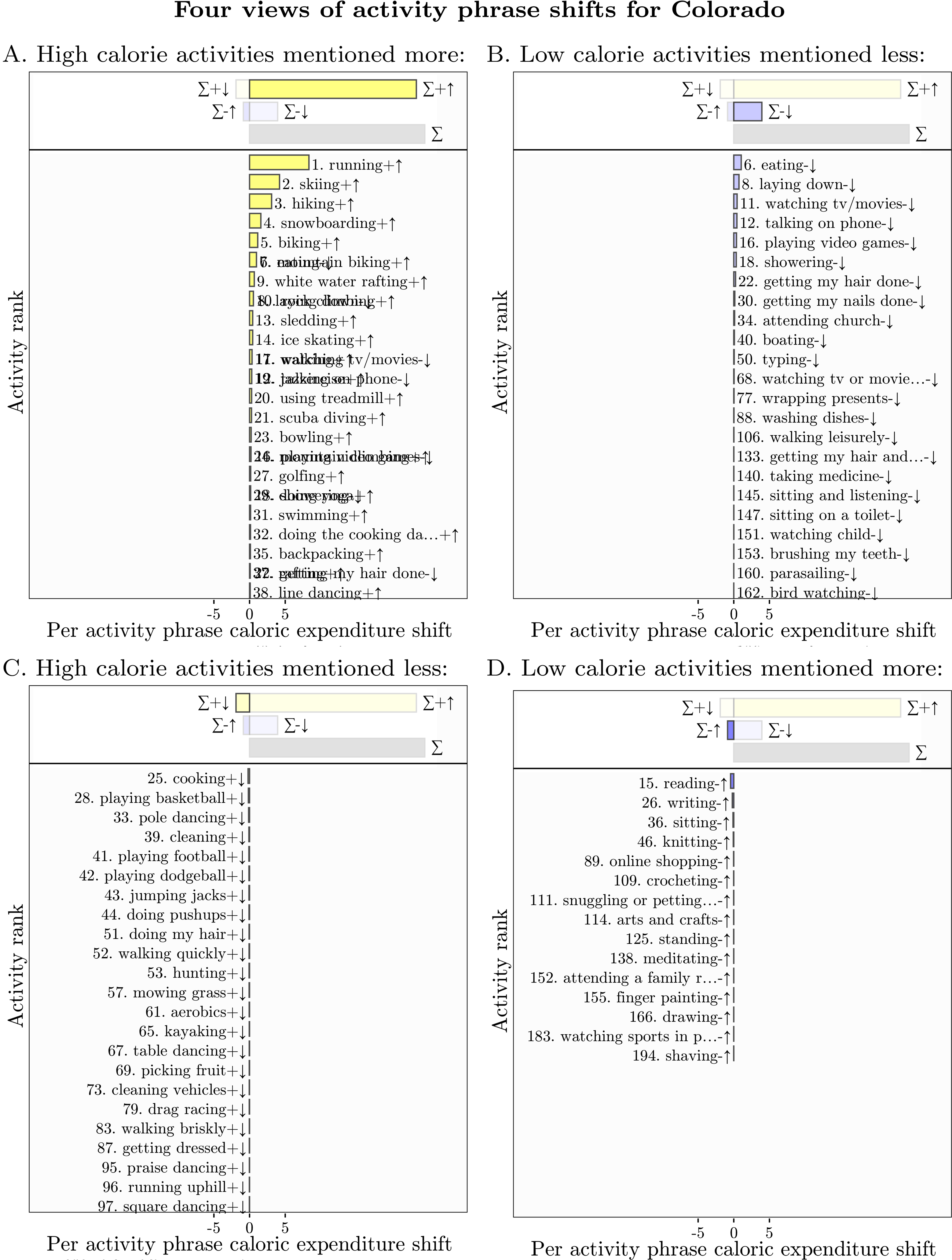

Supplement: S6 Fig — See Fig 6C for the combined shift. See Phrase Shifts in the Analysis and Results section for an explanation of phrase shifts. (TIFF) [file pone.0168893.s006.tiff]

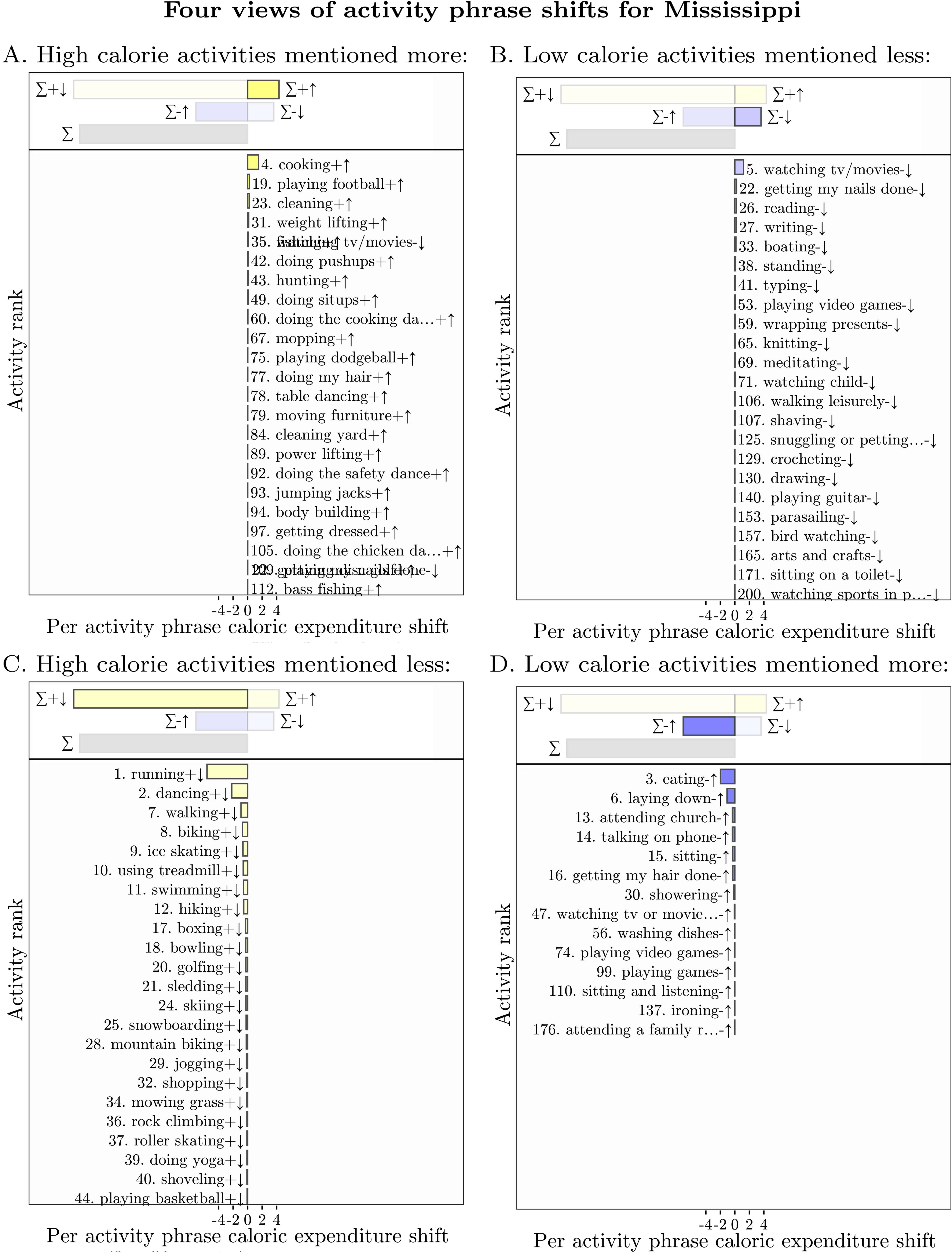

Supplement: S7 Fig — See Fig 6D for the combined shift. See Phrase Shifts in the Analysis and Results section for an explanation of phrase shifts. (TIFF) [file pone.0168893.s007.tiff]
