# Supplementary material for: The Lexicocalorimeter: Gauging public health through caloric input and output on social media
Source: PLoS One. 2017 Feb 10;12(2):e0168893. doi: 10.1371/journal.pone.0168893 (PMC5302853; doi:10.1371/journal.pone.0168893)
Supplement: S1 Table — Spearman correlation coefficients, ρ^s, and Benjamini-Hochberg q-values for caloric input Cin, caloric output Cout, and caloric ratio Crat = Cout/Cin and demographic data related to food and physical activity, Big Five personality traits [31], health and well-being rankings by state, and socioeconomic status, correlated, ordered from strongest to weakest Spearman correlations with caloric ratio. (PDF) [file pone.0168893.s008.pdf]

Health and/or well-being quantity	$\hat{\rho}_s$ for C_{rat}	q-val	$\hat{\rho}_s$ for C_{in}	q-val	$\hat{\rho}_s$ for C_{out}	q-val
**1.** % no physical activity in past 30 days [24]	-0.78	3.07×10^{-09}	0.58	4.91×10^{-05}	-0.66	1.59×10^{-06}
**2.** % have been physically active in past 30 days [24]	0.78	3.07×10^{-09}	-0.58	5.50×10^{-05}	0.67	1.31×10^{-06}
**3.** % high blood pressure [24]	-0.77	3.07×10^{-09}	0.39	1.16×10^{-02}	-0.78	3.07×10^{-09}
**4.** Heart disease death rate [27]	-0.75	1.02×10^{-08}	0.38	1.24×10^{-02}	-0.73	2.07×10^{-08}
**5.** Adult diabetes rate [25]	-0.74	1.17×10^{-08}	0.34	2.77×10^{-02}	-0.77	3.07×10^{-09}
**6.** CNBC quality of life ranking [26]	-0.74	1.87×10^{-08}	0.33	3.22×10^{-02}	-0.77	3.60×10^{-09}
**7.** % adult overweight/obesity [27]	-0.71	1.33×10^{-07}	0.53	3.14×10^{-04}	-0.59	3.56×10^{-05}
**8.** Gallup Wellbeing score [4]	0.7	3.17×10^{-07}	-0.33	3.38×10^{-02}	0.73	4.35×10^{-08}
**9.** % adult obesity [25]	-0.69	3.10×10^{-07}	0.52	4.11×10^{-04}	-0.59	3.56×10^{-05}
**10.** America's Health Rankings, overall [24]	-0.69	1.31×10^{-06}	0.4	9.14×10^{-03}	-0.67	2.65×10^{-06}
**11.** Life expectancy at birth [27]	0.67	7.92×10^{-07}	-0.36	1.59×10^{-02}	0.65	2.58×10^{-06}
**12.** % child overweight/obesity [27]	-0.65	2.58×10^{-06}	0.34	2.82×10^{-02}	-0.64	3.06×10^{-06}
**13.** % who eat fruit less than once a day [28]	-0.65	2.58×10^{-06}	0.57	7.45×10^{-05}	-0.51	5.89×10^{-04}
**14.** % who eat vegetables less than once a day [28]	-0.61	1.32×10^{-05}	0.53	3.14×10^{-04}	-0.46	1.72×10^{-03}
**15.** Median daily intake of fruits [28]	0.59	3.56×10^{-05}	-0.59	3.56×10^{-05}	0.41	5.73×10^{-03}
**16.** Smoking rate [27]	-0.59	3.81×10^{-05}	0.47	1.60×10^{-03}	-0.48	1.24×10^{-03}
**17.** Median daily intake of vegetables [28]	0.5	7.25×10^{-04}	-0.56	1.03×10^{-04}	0.31	4.09×10^{-02}
**18.** Median household income [27]	0.48	1.37×10^{-03}	-0.5	8.58×10^{-04}	0.4	9.07×10^{-03}
**19.** % high cholesterol [24]	-0.48	1.26×10^{-03}	0.24	1.16×10^{-01}	-0.48	1.05×10^{-03}
**20.** Colorectal cancer rate [25]	-0.47	1.72×10^{-03}	0.56	1.37×10^{-04}	-0.27	8.35×10^{-02}
**21.** Brain health ranking [29] (lower is better)	-0.46	1.95×10^{-03}	0.55	1.74×10^{-04}	-0.29	5.43×10^{-02}
**22.** US Census Gini index score [30] (lower is better)	-0.44	3.60×10^{-03}	0.11	5.12×10^{-01}	-0.5	6.22×10^{-04}
**23.** % with bachelor's degree or higher [6]	0.42	4.86×10^{-03}	-0.43	4.21×10^{-03}	0.33	2.82×10^{-02}
**24.** Avg # poor mental health days, past 30 days [24]	-0.39	9.87×10^{-03}	0.1	5.31×10^{-01}	-0.48	1.23×10^{-03}
**25.** Neuroticism Big Five personality trait [31]	-0.37	1.33×10^{-02}	0.23	1.35×10^{-01}	-0.37	1.42×10^{-02}
**26.** Binge drinking rate [24]	0.34	2.91×10^{-02}	-0.12	4.88×10^{-01}	0.41	6.23×10^{-03}
**27.** Farmers markets per 100,000 in pop. [28]	0.33	2.96×10^{-02}	-0.01	9.59×10^{-01}	0.42	5.41×10^{-03}
**28.** Extraversion Big Five personality trait [31]	-0.33	2.83×10^{-02}	0.13	4.13×10^{-01}	-0.29	5.36×10^{-02}
**29.** Avg # poor physical health days, past 30 days [24]	-0.32	3.81×10^{-02}	0.16	3.32×10^{-01}	-0.38	1.16×10^{-02}
**30.** Strolling of the Heifers locavore score (lower is better) [32]	-0.31	4.59×10^{-02}	-0.16	3.32×10^{-01}	-0.45	3.16×10^{-03}
**31.** % schools offering fruit/veg at celebrations [28]	0.25	1.16×10^{-01}	-0.38	1.36×10^{-02}	0.05	7.75×10^{-01}
**32.** Openness Big Five personality trait [31]	0.23	1.31×10^{-01}	-0.42	5.43×10^{-03}	0.04	7.95×10^{-01}
**33.** % cropland harvested for fruits/veg [28]	0.18	2.53×10^{-01}	-0.53	2.90×10^{-04}	-0.04	7.95×10^{-01}
**34.** Conscientiousness Big Five personality trait [31]	-0.1	5.31×10^{-01}	0.14	3.97×10^{-01}	-0.05	7.78×10^{-01}
**35.** % census tracts, healthy food retailer within 1/2 mile [28]	-0.06	7.47×10^{-01}	-0.39	1.09×10^{-02}	-0.24	1.28×10^{-01}
**36.** George Mason overall freedom ranking [33] (lower is freer)	-0.02	8.90×10^{-01}	-0.05	7.73×10^{-01}	-0.1	5.58×10^{-01}
**37.** Agreeableness Big Five personality trait [31]	0	9.95×10^{-01}	0.24	1.26×10^{-01}	0.08	6.41×10^{-01}

S1 Table. Identical to Tab. 1 but with liquids included. Spearman correlation coefficients, $\hat{\rho}_s$, and Benjamini-Hochberg q-values for caloric input C_{in}, caloric output C_{out}, and caloric ratio $C_{rat} = C_{out}/C_{in}$ and demographic data related to food and physical activity, Big Five personality traits [31], health and well-being rankings by state, and socioeconomic status, correlated, ordered from strongest to weakest Spearman correlations with caloric ratio.
