# Supplementary material for: The Lexicocalorimeter: Gauging public health through caloric input and output on social media
Source: PLoS One. 2017 Feb 10;12(2):e0168893. doi: 10.1371/journal.pone.0168893 (PMC5302853; doi:10.1371/journal.pone.0168893)
Supplement: S2 Table — Spearman correlation coefficients, ρ^s, and Benjamini-Hochberg q-values for caloric input Cin, caloric output Cout, and caloric difference Cdiff(α) = αCout + (1 − α)Cin and demographic data related to food and physical activity, Big Five personality traits [31], health and well-being rankings by state, and socioeconomic status, correlated, ordered from strongest to weakest Spearman correlations with caloric ratio. We chose α so that the average of Cout matched the average of αCin. (PDF) [file pone.0168893.s009.pdf]

**Health and/or well-being quantity**	$\hat{\rho}_s$ for C_{diff}	q-**val**	$\hat{\rho}_s$ for C_{in}	q-**val**	$\hat{\rho}_s$ for C_{out}	q-**val**
**1.** % no physical activity in past 30 days [24]	-0.79	1.77×10^{-09}	0.58	5.67×10^{-05}	-0.66	1.51×10^{-06}
**2.** % have been physically active in past 30 days [24]	0.79	1.77×10^{-09}	-0.57	6.53×10^{-05}	0.67	1.24×10^{-06}
**3.** % high blood pressure [24]	-0.78	2.72×10^{-09}	0.32	4.05×10^{-02}	-0.78	2.72×10^{-09}
**4.** Adult diabetes rate [25]	-0.76	5.26×10^{-09}	0.29	6.16×10^{-02}	-0.77	2.73×10^{-09}
**5.** CNBC quality of life ranking [26]	-0.75	8.07×10^{-09}	0.28	7.34×10^{-02}	-0.77	3.60×10^{-09}
**6.** % adult overweight/obesity [27]	-0.73	2.40×10^{-08}	0.55	1.41×10^{-04}	-0.59	3.07×10^{-05}
**7.** Heart disease death rate [27]	-0.73	2.07×10^{-08}	0.34	2.82×10^{-02}	-0.73	2.07×10^{-08}
**8.** Gallup Wellbeing score [4]	0.73	3.83×10^{-08}	-0.31	4.43×10^{-02}	0.73	3.70×10^{-08}
**9.** % adult obesity [25]	-0.72	3.70×10^{-08}	0.53	2.26×10^{-04}	-0.59	2.94×10^{-05}
**10.** America's Health Rankings, overall [24]	-0.72	3.93×10^{-07}	0.43	4.74×10^{-03}	-0.67	2.77×10^{-06}
**11.** Life expectancy at birth [27]	0.68	4.27×10^{-07}	-0.4	6.91×10^{-03}	0.65	2.64×10^{-06}
**12.** % who eat fruit less than once a day [28]	-0.67	9.44×10^{-07}	0.61	1.38×10^{-05}	-0.51	5.23×10^{-04}
**13.** % child overweight/obesity [27]	-0.64	3.03×10^{-06}	0.27	7.55×10^{-02}	-0.64	3.06×10^{-06}
**14.** % who eat vegetables less than once a day [28]	-0.61	1.38×10^{-05}	0.51	5.21×10^{-04}	-0.46	1.57×10^{-03}
**15.** Median daily intake of fruits [28]	0.6	1.68×10^{-05}	-0.62	8.33×10^{-06}	0.41	5.44×10^{-03}
**16.** Smoking rate [27]	-0.6	2.14×10^{-05}	0.51	5.19×10^{-04}	-0.48	1.08×10^{-03}
**17.** Median household income [27]	0.51	5.19×10^{-04}	-0.53	3.27×10^{-04}	0.4	8.38×10^{-03}
**18.** Median daily intake of vegetables [28]	0.5	5.72×10^{-04}	-0.56	7.44×10^{-05}	0.31	4.36×10^{-02}
**19.** Brain health ranking [29] (lower is better)	-0.5	7.50×10^{-04}	0.62	1.38×10^{-05}	-0.29	5.70×10^{-02}
**20.** % high cholesterol [24]	-0.49	7.88×10^{-04}	0.23	1.45×10^{-01}	-0.48	9.05×10^{-04}
**21.** % with bachelor's degree or higher [6]	0.47	1.48×10^{-03}	-0.54	1.66×10^{-04}	0.33	2.82×10^{-02}
**22.** Colorectal cancer rate [25]	-0.44	3.82×10^{-03}	0.53	3.59×10^{-04}	-0.27	8.25×10^{-02}
**23.** US Census Gini index score [30] (lower is better)	-0.42	4.99×10^{-03}	-0.03	8.45×10^{-01}	-0.5	5.55×10^{-04}
**24.** Avg # poor mental health days, past 30 days [24]	-0.42	5.44×10^{-03}	0.12	4.75×10^{-01}	-0.48	1.06×10^{-03}
**25.** Neuroticism Big Five personality trait [31]	-0.38	1.13×10^{-02}	0.2	2.03×10^{-01}	-0.37	1.42×10^{-02}
**26.** Binge drinking rate [24]	0.38	1.32×10^{-02}	-0.15	3.56×10^{-01}	0.41	5.84×10^{-03}
**27.** Avg # poor physical health days, past 30 days [24]	-0.35	2.34×10^{-02}	0.19	2.19×10^{-01}	-0.38	1.13×10^{-02}
**28.** Farmers markets per 100,000 in pop. [28]	0.33	2.82×10^{-02}	0.06	7.17×10^{-01}	0.42	5.05×10^{-03}
**29.** Strolling of the Heifers locavore score (lower is better) [32]	-0.29	6.44×10^{-02}	-0.3	5.41×10^{-02}	-0.45	2.94×10^{-03}
**30.** Extraversion Big Five personality trait [31]	-0.28	6.89×10^{-02}	0.03	8.50×10^{-01}	-0.29	5.63×10^{-02}
**31.** % schools offering fruit/veg at celebrations [28]	0.24	1.26×10^{-01}	-0.46	1.96×10^{-03}	0.05	7.90×10^{-01}
**32.** Openness Big Five personality trait [31]	0.24	1.26×10^{-01}	-0.5	6.11×10^{-04}	0.04	8.10×10^{-01}
**33.** % cropland harvested for fruits/veg [28]	0.19	2.35×10^{-01}	-0.62	1.37×10^{-05}	-0.04	8.10×10^{-01}
**34.** Conscientiousness Big Five personality trait [31]	-0.12	4.62×10^{-01}	0.2	2.10×10^{-01}	-0.05	7.93×10^{-01}
**35.** % census tracts, healthy food retailer within 1/2 mile [28]	-0.02	8.86×10^{-01}	-0.52	3.68×10^{-04}	-0.24	1.28×10^{-01}
**36.** George Mason overall freedom ranking [33] (lower is freer)	-0.02	8.88×10^{-01}	-0.11	5.15×10^{-01}	-0.1	5.64×10^{-01}
**37.** Agreeableness Big Five personality trait [31]	-0.01	9.42×10^{-01}	0.22	1.50×10^{-01}	0.08	6.47×10^{-01}

S2 Table. Identical to Tab. 1 but using a caloric difference rather than caloric ratio. Spearman correlation coefficients, $\hat{\rho}_s$, and Benjamini-Hochberg q-values for caloric input C_{in}, caloric output C_{out}, and caloric difference $C_{diff}(\alpha) = \alpha C_{out} + (1 - \alpha) C_{in}$ and demographic data related to food and physical activity, Big Five personality traits [31], health and well-being rankings by state, and socioeconomic status, correlated, ordered from strongest to weakest Spearman correlations with caloric ratio. We chose α so that the average of C_{out} matched the average of αC_{in}.
